# The hidden waves in the ECG uncovered revealing a sound automated interpretation method

**DOI:** 10.1038/s41598-021-82520-w

**Published:** 2021-02-12

**Authors:** Cristina Rueda, Yolanda Larriba, Adrian Lamela

**Affiliations:** Department of Statistics and Operations Research, Universidad de Valladolid, Valladolid, Spain

**Keywords:** Computational biology and bioinformatics, Physiology, Cardiology, Medical research

## Abstract

A novel approach for analysing cardiac rhythm data is presented in this paper. Heartbeats are decomposed into the five fundamental *P*, *Q*, *R*, *S* and *T* waves plus an error term to account for artifacts in the data which provides a meaningful, physical interpretation of the heart’s electric system. The morphology of each wave is concisely described using four parameters that allow all the different patterns in heartbeats to be characterized and thus differentiated This multi-purpose approach solves such questions as the extraction of interpretable features, the detection of the fiducial marks of the fundamental waves, or the generation of synthetic data and the denoising of signals. Yet the greatest benefit from this new discovery will be the automatic diagnosis of heart anomalies as well as other clinical uses with great advantages compared to the rigid, vulnerable and black box machine learning procedures, widely used in medical devices. The paper shows the enormous potential of the method in practice; specifically, the capability to discriminate subjects, characterize morphologies and detect the fiducial marks (reference points) are validated numerically using simulated and real data, thus proving that it outperforms its competitors.

## Introduction

**Figure 1:**
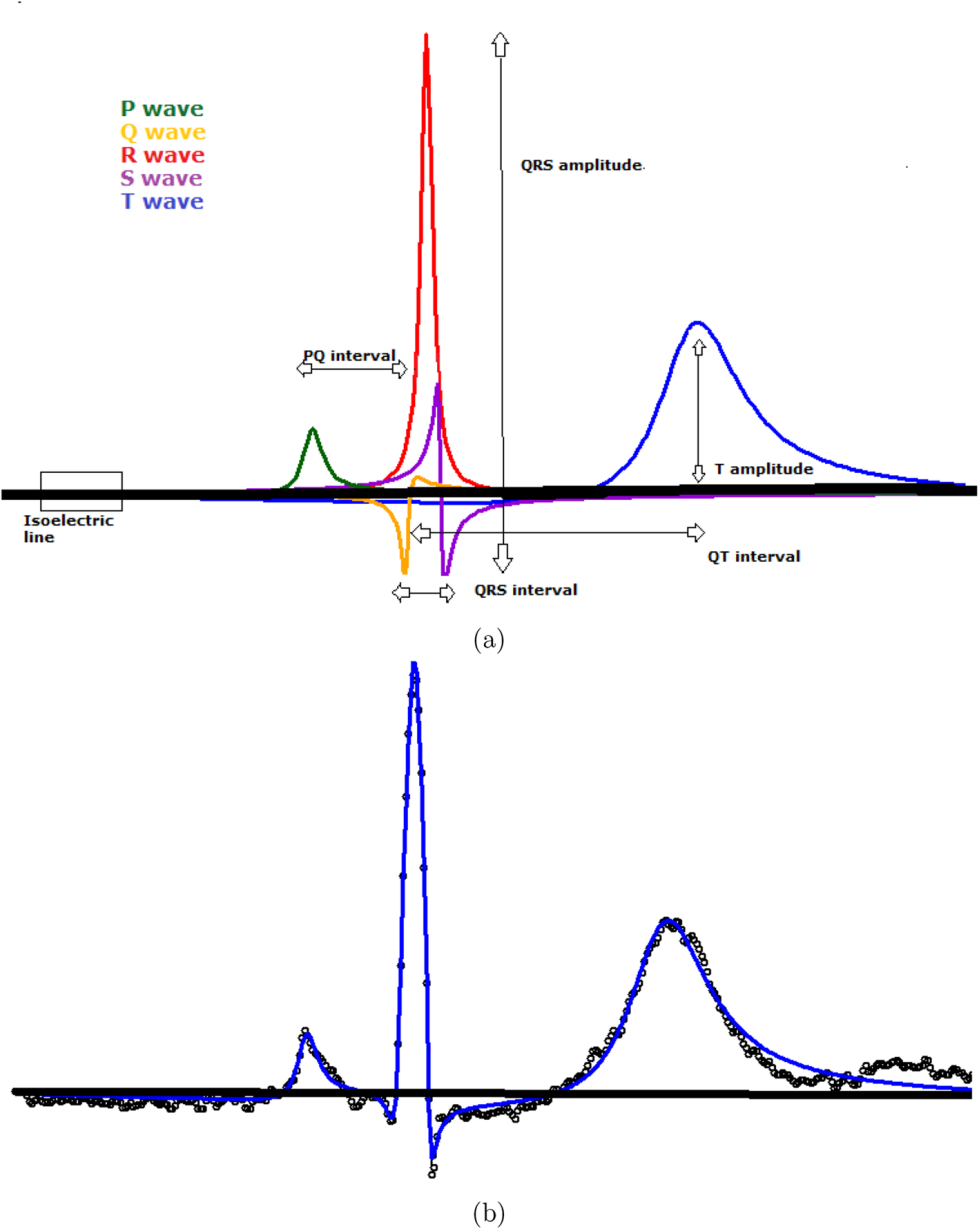
(**a**) The five waves: *P*, *Q*, *R*, *S*, *T* derived from the $FMM_{ecg}$ model and some of the main features that are derived from the parameters of the model in a simple way. (**b**) Observed signal (black points) and $FMM_{ecg}$ fit (blue). Data from patient *sel*106 from MIT-BIT Arrhythmia Database from Physionet (http://www.physionet.org).

The importance of the ECG signal in diagnosis and prediction of cardiovascular diseases is worth noting. The process recorded in the ECG is the periodic electrical activity of the heart. This activity represents the contraction and relaxation of the atria and ventricle, processes related to the crests and troughs of the ECG waveform, labelled *P*, *Q*, *R*, *S* and *T* (see Fig. 1(a)). The main features used in the medical practice are related to the location and amplitudes of these waves. A standard ECG signal is registered using twelve leads calculated from different electrodes being lead II the reference one.

The mere visual observation of the ECG signals, although made by a consolidated expert, is not enough to discover the diversity of abnormalities and the specific characteristics of the morphology of each ECG. Moreover, it requires an enormous amount of human expertise resources. Therefore, a rigorous automatic analysis of digitalized ECG signals can be of great help. However, although it has been a question that has received a lot of attention in the literature over the last decades, there is still no suitable mathematical model or computational approach, that accurately describes the spectrum of morphologies in ECG signals, as is noted in recent references on this topic, such as^1–4^ or^5^ among others.

The literature addressing the problem of the automatic interpretation of the ECG is so extensive that it is difficult to include a complete review here. The most widely used model-based approach describes the main waves with a combination of basic functions, the Gaussians being the preferred ones, for a single or average beat. A precursor model was proposed by^6^ and was more recently considered by^7^ or^8^ among others, whom proposed improvements in the formulation and estimation algorithms^9^. Also recently uses this approach for the predictive modelling of drug effects on ECG signals. These approaches have important shortcomings. In particular, the Gaussian functions fail to reproduce the morphology of the waves in a simple way, especially for atypical and noisy ECGs, where the complexity as well as the risk of overfitting increase. Moreover, most of the parameters do not have a specific morphological meaning. Other examples of model-based proposals are those by^10–13^ or^14^. These approaches may be suitable to study some specific questions, but, they are far from being multi-purpose methods.

However, many of the recent papers are contributions to computational and machine learning approaches. Some of the large list of references are:^15–19^ or^20^. Also, the papers by^4,21–23^ and^24^ extended the list of procedures and their pros and cons for the automatic analysis of ECGs. In general, machine learning approaches success is very dependent on the training set, the selection of diagnostic groups, the preprocessing and the database, see^25,26^ or^27^ among others. Furthermore, they are rigid and black-box procedures that are susceptible to adversial attacks^28^.

The approach, called $FMM_{ecg}$, presented in this paper is just the opposite.

This novel approach combines a physically meaningful formulation with good statistical and computational properties. $FMM_{ecg}$ is a multicomponent model, where each component is a single *FMM* (Frequency Modulate Möbius) oscillator and specific ECG parameter restrictions are included. Single *FMM* models are recently proposed by^29^ to predict oscillatory signals in several different fields from biology to astrophysics. The distinguishing feature of the *FMM* model is that it is formulated in terms of the phase, which is the angular variable that represents the periodic movement of the oscillation. Specifically, the $FMM_{ecg}$ model is defined as the combination of exactly five oscillatory components referred to as waves: $W_J(), J = P, Q, R, S, T$, which correspond to the fundamental waves in a heartbeat; plus an error term that accounts for artifacts in the data. Four parameters characterize each wave and, a Maximization-Identification (MI) algorithm is designed to estimate them. This algorithm alternates, iteratively, between a maximization M-step and a wave-identification I-step. While the model proposal is valid for signals registered elsewhere, the I-step is lead-specific. Nevertheless, the I-step can be easily adapted to signals registered in other regions.

The main virtues of the novel approach can be summarized in five points which are validated in the paper. Firstly, the $FMM_{ecg}$ model is physically meaningful representing the conduction of the electrical signal by the combination of five main waves presented in a normal heartbeat. Therefore, alterations in a specific wave identify the part of the heart responsible. Secondly, for each wave, four parameters are extracted, measuring, amplitude, location, scale and shape. These parameters are able to characterize, reproduce and identify the variety of morphologies observed in real ECG signals. In addition, other interesting features are easily derived from these main parameters. Thirdly, the MI algorithm provides accurate and robust estimates of the model parameters discarding overfitting problems. Fourthly, the approach is not dependent on a training set and is valid for any ECG registered signal, independently of the preprocessing, frequency or scale. Finally, the approach has strong theoretical properties: is maximum likelihood based while assuming Gaussian errors, the parameters are identifiable and the estimators are consistent.

The most exciting questions shown in the paper are that $FMM_{ecg}$ model describes a wide variety of ECG signals with high accuracy, eliminating noise artifacts and that the $FMM_{ecg}$ parameters are interpretable features describing the morphology of ECG signals and solve specific questions such as the detection of fiducial marks or discrimination among patterns. The important question of biometric identification is also partially addressed in this paper.

The validation of the $FMM_{ecg}$ approach is not simple as there is no multi-purpose approach in the literature similar to $FMM_{ecg}$. Therefore, the main properties of $FMM_{ecg}$ are validated considering diverse alternative approaches. On the one hand, for global goodness of fit consistency, robustness and discriminative power, the $FMM_{ecg}$ is compared with a model-based approach, which considers a combination of Gaussian components, similar to that proposed by^8^. On the other hand, the ability to detect fiducial marks is compared with several recent machine learning approaches, in particular, those considered by^18^. In this paper, we deal with signals from lead II and close to it. Simulated and publicly available data from databases in Physionet (www.physionet.org)^30^ are used. Very promising results have been obtained from real data. For example, Figure 1 shows the result of applying the $FMM_{ecg}$ to data from patient *sel106* in MIT-BIT database, a representative, typical pattern used by many authors. The waves drawn in Figure 1 (a) have not been artificially generated, but are simply the estimators provided by the MI algorithm for the five waves: $W_J (), J = P, Q, R, S, T$. While panel (b) shows the combined $FMM_{ecg}$ fit.

## Results

### Overview of the $FMM_{ecg}$ model

Data from one heartbeat are analysed as a sum of five waves plus an intercept parameter, *M*, and an error term.

Each of the individual wave is described with four parameters: $(A,\alpha ,\beta ,\omega )$. The parameter *A* measures the wave amplitude; a zero value indicating that the corresponding wave is not present. The parameter $\alpha$ is a location parameter. In addition, $\beta$ and $\omega$ measure skewness and kurtosis, respectively; and they are useful to describe the shape of the waves, in particular if they are crest or troughs. More specifically, assuming $\alpha =0$, the values for parameter $\beta$ close to $\pi$ (or $2\pi$) represent a unimodal symmetric wave (or an inverse unimodal symmetric wave); as $\beta$ moves away from these values, the patterns are more asymmetric and the values of $\beta$ equal to $\pi /2$ or $3\pi /2$ describe a wave with both crest and trough with completely asymmetric patterns. The parameter $\omega$ measures the sharpness of the peak, $\omega =1$ corresponds to an exact sinusoidal shape and, as $\omega$ approaches zero, the sharpness becomes more pronounced (see^29^ for more detail in parameter interpretation).

Other wave features extracted from the main parameters are the crest and trough times, these marks denoted by $t^U$ and $t^L$ respectively.

Moreover, measurements of inter-wave intervals, as those in Fig. 1a are calculated using distances between these marks, and other features, such as those used in the literature of ECG interpretation, can be easily derived from the main parameters. However, while the estimation of features proposed in the literature often depends on the algorithm and voltage measurements^4^, $FMM_{ecg}$ provides systematic and reliable measurements.

In the estimation process, to improve the waves identification when atypical patterns are observed, additional conditions are imposed.

### Validation

Three different validation analyses have been performed. The first two refer to the QT database^31^ and the third is a simulation experiment, which is deferred to Supplementary Information. In particular, six characteristic patterns of different pathologies plus one typical pattern, have been simulated (Figure S1). The analysis of simulated beats validates the global fit of the model (Table S1), the accuracy and consistency of the parameters’ estimators (Table S2 and Figure S2) and, the potential to detect fiducial marks (Table S3). Finally, the potential of $FMM_{ecg}$ features to discriminate among patterns is revealed as a Fisher linear discriminant analysis (LDA) with the one-leave-out rule which perfectly discriminates the seven simulated patterns (Subsection 1.4.1 in the Supplementary Information).

On the other hand, the QT database’s election for the patient identification analysis is because it includes patients from MIT BIH Arrhythmia database, which is a benchmark, and many others from different sources. Moreover, it has been recently used by several authors and provides a wide range of morphologies associated with healthy and pathological ECGs. The database contains signals from two leads. We analyse the segment for each patient for whom the *T* or *P* waves have been manually annotated, as well as the data corresponding to the signal closest to lead II (in most cases it corresponds to the first signal). For patient *sel*42, data from the first signal are not reliable, instead, the inverse of the second one is analysed as it represents a signal closer to lead II.

A total of 3,623 single beats signals have been analysed.

**Figure 2:**
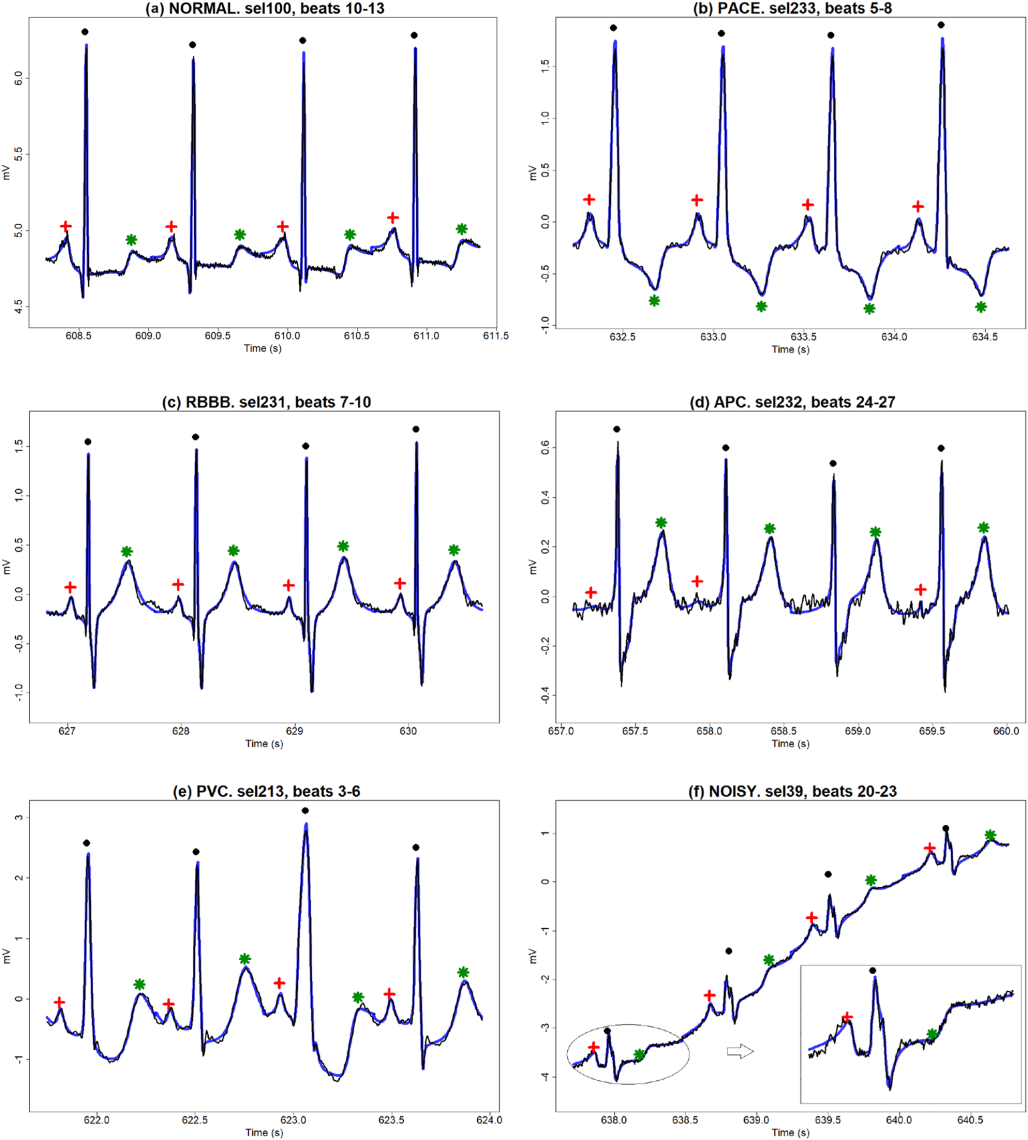
Observed ECG segments (black lines), $FMM_{ecg}$ fits (blue lines) and fiducial marks for *R* wave ($\bullet$), *T* wave ($\star$), *P* wave ($+$); for (**a**) NORMAL, (**b**) PACE, (**c**) RBBB, (**d**) APC, (**e**) PVC and (**f**) NOISY patterns.

#### Analysis of QT database signals: graphical and analytical results

For each single beat, the value of a coefficient of determination, $R^2$, that measures the proportion of the variance explained by the model, out of the total variance, is calculated.

The $R^2$ values are very high across patients, being $R^2$ global mean (SD) equal to 0.98(0.02).

Figure 2 illustrates the model performance on six ECG segments from different patients. The first five correspond to the most frequent patterns according to Physionet’s classification of the heartbeats by their morphology. They are labelled as NORMAL (patient *sel100*, typical pattern), PACE (patient *sel233*, Paced beat), RBBB (patient *sel231*, Right bundle branch block beat), APC (patient *sel232*, Atrial premature beat), and PVC (patient *sel213*, Premature ventricular contraction); besides a NOISY pattern (patient *sel39*) is also considered. The NOISY segment exhibits both, low and high frequency noise as the zoom in the corresponding plot shows.

**Figure 3:**
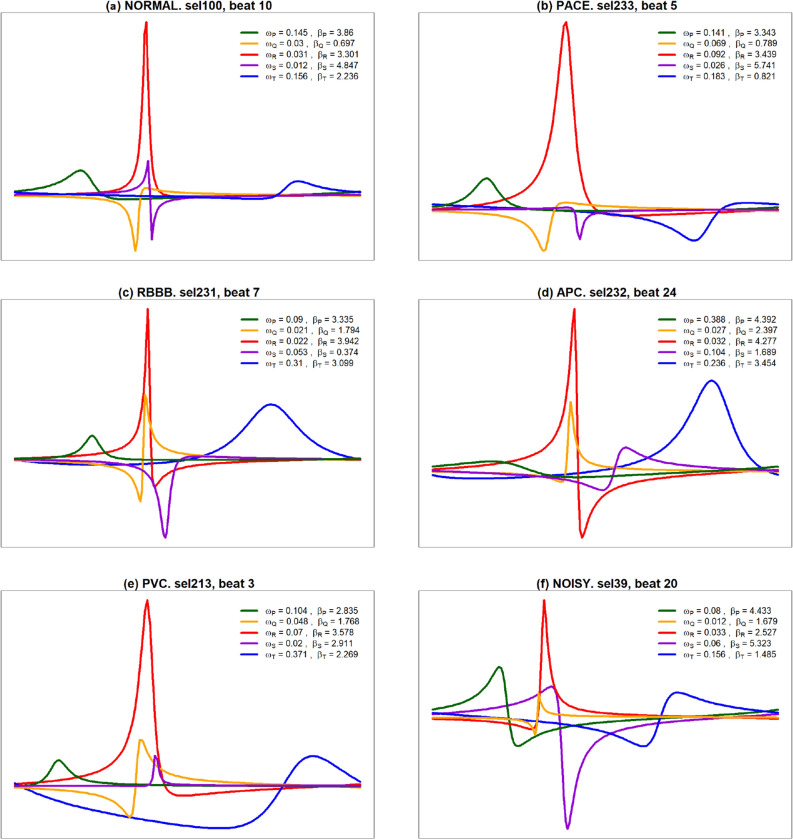
$FMM_{ecg}$ waves and corresponding parameters, for representative beats from (**a**) NORMAL, (**b**) PACE, (**c**) RBBB, (**d**) APC, (**e**) PVC and (**f**) NOISY patterns. *P* (green), *Q* (yellow), *R* (red), *S* (violet), and *T* (blue).

It is interesting to observe how the specific shapes of the five main waves contribute to draw the observed pattern of the different morphologies as it is shown in Fig. 3. The estimated values of the parameters, recorded on the right side of the plots, quantify and describe the patterns, and explain the differences between the morphologies.

The accuracy of $FMM_{ecg}$ to extract ECG local waves can be measured with the percentile interval amplitudes given, for the main parameters of each of the 105 QT patients, in Table S4.

On the other hand, the potential of the $FMM_{ecg}$ parameters to solve the problem of subject identification is also shown. A Fisher LDA is applied, using as predictors: $A_J, \omega _J, \beta _j; J=P,Q,R,S,T$ (where missing values are replaced with the median value of the corresponding patient) and the one-leave-out rule to estimate the error rate which is 8.6%. Considering the difficulty of the task to discriminate among 105 classes, some of them with apparently very similar ECG patterns, an error rate of 8.6% is quite good. The comparison with other studies in the literature is not feasible since the selection of heartbeats, patients, or the classes to discriminate differs from one paper to another, in many of them, the choice seems to be made ad hoc. As far as we know, this is the first time that this milestone has been achieved for the QT database, since other authors consider specifically selected sets of patients of a much smaller size (see^27,32^ and references therein). Moreover, a complete analysis is provided in the Supplementary Information including, specific-patient plots and statistics for the main $FMM_{ecg}$ parameters. Separately, by QT subgroups defined by the source of the data, Fisher LDA analyses have been performed. Confusion matrices are shown.

The results reveal the consistency and reliability of estimators and great potential for individual identification tasks.

#### Analysis of QT database signals: *P* and *T* wave annotations

This question is still a challenge as^33–35^ or^36^, among others, confirm.

Let $t_J^{FI}$, $J=T,P$ be the fiducial $FMM_{ecg}$ marks. Where if wave *J* is positive ($t_J^{FI}=t_J^{U}$) or negative ($t_J^{FI}=t_J^{L}$) is determined by the parameters of the model.

In order to perform a fair comparison with alternatives approaches, we follow the analysis in^18^. There are several reasons to consider this as the reference paper. First, the aim of the paper is specifically the detection of *P* and *T* waves; second, the same 105 QT patients are analysed; third, it clearly specifies how the false positives and false negatives are determined, which facilitates a fair comparison; and finally, it includes the results of many previous studies and combination of approaches, which allow us to compare $FMM_{ecg}$ approach against an important collection of alternative proposals.

Several measures are calculated to assess the wave detection that are described in the Methods Section, sensitivity (*Se*), positive predictive value (*PPV*), detection error rate *DER* and the *F*1 score.

Table 1 shows the results, along with the four best methods in^18^, i.e., Martinez PT, Martinez WT+templates, Martinez WT+PT and Martinez PT + templates.

**Table 1:** Summary of performance measures *P* and *T* waves detection from QT first signal data.

Method	No. beats	*P* Wave						
		*TP*	*FP*	*FN*	*Se* (%)	*PPV* (%)	*DER* (%)	*F1* (%)
$FMM_{ecg}$	3194	3085	212	109	**96**.**59**	**93**.**57**	**10**.**05**	**95**.**05**
Martinez PT	3194	2859	342	335	89.51	89.32	21.20	89.41
Martinez WT+templates	3194	2751	395	443	86.13	87.44	26.23	86.78
Martinez WT+PT	3194	2932	416	262	91.80	87.57	21.23	89.64
Martinez PT+templates	3194	2816	320	378	88.15	89.90	21.85	89.41

Method	No. beats	*T* Wave						
		*TP*	*FP*	*FN*	*Se* (%)	*PPV* (%)	*DER* (%)	*F1* (%)
$FMM_{ecg}$	3542	3542	415	0	**100**	**89**.**51**	**11**.**72**	**94**.**47**
Martinez PT	3542	2985	559	557	84.27	84.22	31.50	84.25
Martinez WT+templates	3542	3115	464	427	87.94	87.03	25.15	87.49
Martinez WT+PT	3542	3030	558	512	85.54	84.44	30.20	84.99
Martinez PT+templates	3542	3035	505	507	85.68	85.73	28.57	85.71

Bold values indicate the highest values for the measures.

$FMM_{ecg}$ gives the best results for all the validation measures and for both *P* and *T* mark detection. It is especially striking that DER is less than halved in comparison to other methods for both *T* and *P* wave detection. The accurate detection of waves provided by $FMM_{ecg}$ is more valuable as the algorithm has not been specifically designed for this task, as it also serves other purposes.

Specific patient measures are given in Tables S5 and S6. Besides, Figures S11–S16 show cases where the $FMM_{ecg}$ annotation is correct but is annotated mistaken as *FN* or *FP*. In some of those cases, what happens is that Physionet annotation uses the information from the second signal or from a close beat. In other cases, what happens is the $FMM_{ecg}$ annotation is more reasonable than, or as least as reasonable as, the Physionet annotation, although different. These cases indicate that the good $FMM_{ecg}$ results from Table 1 could even be improved.

## Discussion

From the methodological point of view, two novel contributions are proposed in this paper. On the one hand, a regression model with multiple oscillatory components, which is formulated in terms of angular variables that represent the periodic movement of the waves, and that incorporates restrictions among the parameters, is considered. And, on the other hand, an MI original algorithm of estimation is designed. These methodological contributions have been proved here to be very relevant for their applications in the description of the cardiac rhythm, but the potential is higher as they will likely be able to solve problems in other fields.

As for the contributions to the automatic diagnosis of cardiovascular diseases and other clinical uses, the highlight of our approach is that it provides a set of new parameters and features with high descriptive potential which provides a concise analytical description of the morphology of the five main waves; specifically, its high capacity in human recognition has been demonstrated. Moreover, it is also very reliable, even in abnormal and poor quality ECGs; it does not use training data and it works independently of preprocessing, scale and frequency.

The $FMM_{ecg}$ parameters can be very useful to generate an automatic diagnostic by imitating the recognition skills of human beings, because estimated values under a given condition can be compared with reference values. In addition, the influence of such factors as age, gender, physical condition, medication, anatomic or genetic differences can be taken into account. In fact, actual automatic diagnosis proposals fail due to two main causes; firstly, because different and unreliable measurements are used; secondly, because different problems in origin generate partially similar morphologies and, conversely, a certain anomaly is not associated with a single pattern. Using personalized reference ranges avoids false positives in diagnosis and subscribes to the global trend towards personalized medicine.

Moreover, the new parameters can be used in experimental assays to test medical and preventive strategies, to study the evolution of the heart’s functioning and could even allow inferring personal identity, as well as circumstances as the emotional state at the time of data collection. The important question of biometric identification using ECG features has received attention recently in the literature (see^17,25,26,32,37,38^ among others). The reduced error rate when the 105 QT patients are discriminated is a piece of evidence of the potential of $FMM_{ecg}$ parameters in biometric identification. Nevertheless, ECG morphology changes over time and circumstances and, $FMM_{ecg}$ parameter estimators change consequently. It would be interesting and will be part of the future work, to characterize parameter changes due to time and circumstances and differentiate from random artifacts. Hopefully, in many situations where electrode noise, electronic noise and, other artifact deriving in random noise are being recorded in the ECG signal, the $FMM_{ecg}$ parameters do not change significantly as is shown in the simulation analysis.

The limitations of the approach, which are also challenges and extensions for future research, are sketched out next.

Firstly, a catalog of interesting patterns together with their parametric characterization must be elaborated in collaboration with an expert. This question is partially addressed here, but a much more precise and detailed study is needed. This task should be done by the incorporation of identification algorithms from other leads.

Secondly, there are a few patterns, such as the Atrial Flutter, that do not fit well into the five main wave paradigm, but for which it is possible to design a specific algorithm. The analysis of multiple leads would also facilitate the wave identification task and provide more accurate results.

Finally, the incorporation of covariates, the definition of multivariate models and dynamic models, are statistical extensions to be studied that have several applications in the clinic. Specifically, the covariates would serve to assess the influence of medication or the effect of interventions and multivariate and dynamic models would serve to describe spatio-temporal behaviors and model relationships between biological processes.

## Methods

Suppose $X(t_i), t_1<\cdots <t_n$ are observations from one beat. Without loss of generality, we assume that $t_i\in [0,2\pi ]$ (in any other case, transform the observed time points as in^29^).

For $J \in \lbrace P,Q,R,S,T\rbrace$, let $\upsilon _J=(A_J,\alpha _J,\beta _J,\omega _J)'$ be the four-dimensional parameters describing the waveforms in such a way that

$$\begin{aligned} W_J(t,\upsilon _J)=A_J\cos (\beta _J+2\arctan (\omega _J\tan (\frac{t-\alpha _J}{2}))) \end{aligned}$$

Then, the $FMM_{ecg}$ model, is defined as a parametric additive signal plus error model as follows:

### **Definition 1.**

$$FMM_{ecg}$$ *model* . For $$i=1,\ldots ,n$$:

$$\begin{aligned} X(t_i)= \mu (t_i,\theta )+ e(t_i); \end{aligned}$$

where,

$$\begin{aligned} \mu (t,\theta )= M+\sum _{J\in \lbrace P,Q,R,S,T \rbrace } W_J(t,\upsilon _J); \end{aligned}$$

and

- $$\theta =(M,\upsilon _P,\upsilon _Q.\upsilon _R,\upsilon _S,\upsilon _T)$$ verifying:
  1. $$ M \in \mathfrak {R}$$
  2. $$\upsilon _J \in \mathfrak {R}^+ \times [0,2\pi ] \times [0,2\pi ] \times [0,1]$$, $$J \in \lbrace P,Q,R,S,T \rbrace $$
  3. $$\alpha _P \le \alpha _Q \le \alpha _R \le \alpha _S \le \alpha _T \le \alpha _P $$
- $$(e(t_1),\ldots ,e(t_n))' \sim N_n(0,\sigma ^2I)$$

The incorporation of circular order restrictions among the $\alpha$’s represent the ordered movement of the stimulus from the sinus node to the ventricles, passing through the atria, this giving the model physical interpretability. The restrictions guarantee the identifiability of the parameters once main wave *R* is located.

The fiducial marks are defined for $J\in \lbrace P,Q,R,S,T \rbrace$ as follows:

$$\begin{aligned} t^U_J=\alpha _J+2 \arctan (\frac{1}{\omega _J}\tan (\frac{-\beta _J}{2})); t^L_J=\alpha _J+2 \arctan (\frac{1}{\omega _J}\tan (\frac{\pi -\beta _J}{2})) \end{aligned}$$

In the estimation process, to improve the waves identification when atypical patterns are observed, additional conditions are imposed.

Despite the four specific $FMM_{ecg}$ parameters for each wave, the information about the distance between waves is easily derived using the distance between the $\alpha$’s, by instances, $d(\alpha _Q, \alpha _S)=1-\cos (\alpha _Q-\alpha _S)$ is a measure of the *QRS* duration. Distance measures between any pair of waves can also be defined in a similar way. In fact, the α parameters are used to identify each of the *P*, *Q*, *R*, *S* and *T* waves in the algorithm. Alternatively, the fiducial marks associated with the waves can be considered to derive Euclidean distance measurements. Other interesting theoretical properties and applications of the $FMM_m$ model, a generalization of $FMM_{ecg}$, are described in^39^.

### Estimation algorithm

The application of our method for the QT database analysis and simulations assumes that *QRS* annotations are provided. The detection of the *QRS* complex is a highly researched problem and well solved; interesting references on the subject are^40–43^ and^44^, among others. The *QRS* annotations and *RR* values (distances between consecutive *QRS* annotations), provided by Physionet, are used to select the specific segment corresponding to a single beat in our data analysis. For a given *QRS* annotation, $t^{QRS}$, let $RR_-$ and $RR_+$ be the *RR* obtained from the previous and the next *QRS* annotation, respectively. Therefore, periodicity changes are detected with changes in *RR* values and, the algorithm is adapted to that in such a way that the morphological characteristics of local waves are described regardless of the beat length. Then, the input for the analysis of a single beat are the observations, $X(t_i)$, where $t_i \in [t^{QRS}-40\%RR_-,t^{QRS}+60\%RR_+], i=1,\ldots ,n$, which before entering the algorithm, pass a trend removal step to reduce the influence of the low frequency noise, if necessary.

The MI algorithm, described below, uses these input data to derive predicted values for the voltage and features.

**Figure 4:**
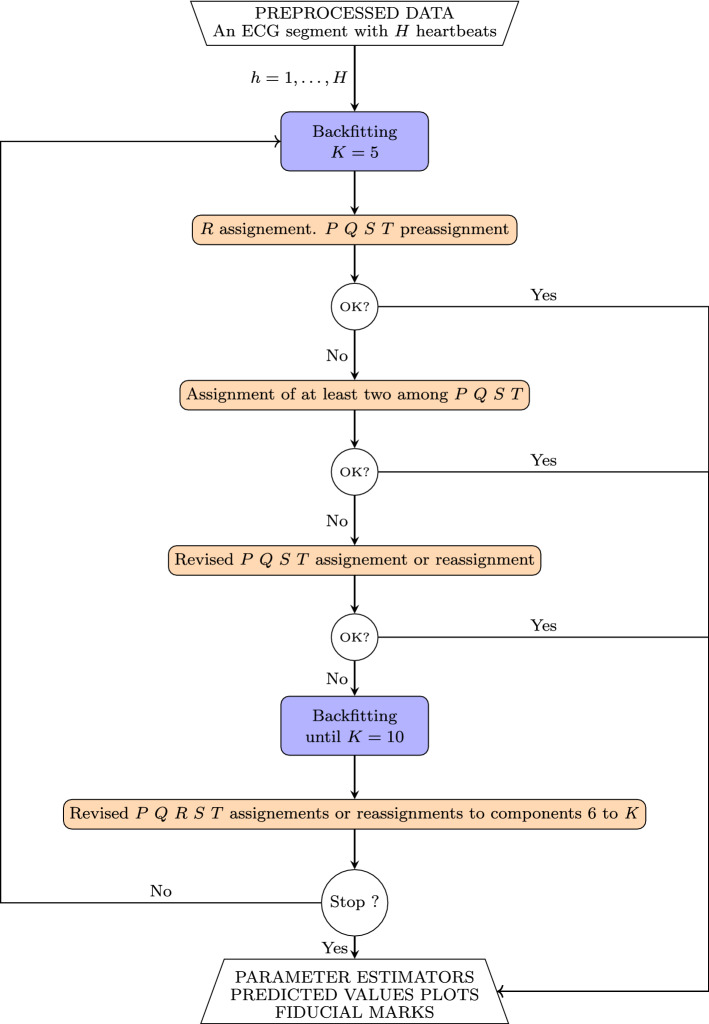
$$FMM_{ecg}$$ MI algorithm.

Consider the model in Definition 1. The estimation problem reduces to solving the following optimization problem:

$$\begin{aligned} Min_{ \theta \in \Theta } \sum _{i=1}^n [ X(t_i)-\mu (t_i,\theta )]^2 \end{aligned}$$

where $\Theta$ is the parametric space. For a typical ECG pattern $\Theta$ is simply defined as in Definition 1 through the restrictions among the $\alpha$’s. However, in order to arrive to a right identification of letters in atypical patterns in real practice, additional restrictions are needed. Mathematically, it means that $\Theta$ is reduced and are incorporated as thresholds in the algorithm.

The optimization problem above is computationally intensive and it is solved using an iterative algorithm which alternates M and I steps that provide successive estimators for $$W_J,J=P,Q,R,S,T$$. The M step provides $$K \ge 5$$ oscillatory components using a backfitting algorithm and the I step assigns $$K \le 5$$ letters to, at most, five of these components. Typically, $$K=5$$, however, in the presence of significant noise or when the morphology is pathological, sometimes, the interesting waves may be null or be hidden between the sixth or seventh component (very exceptionally in others). For each component, the *FMM* parameter values and percentage of explained variance, $$PV $$, are computed. The latter defined as follows,

$$\begin{aligned} PV_k= R^2_{1,\ldots ,k}- R^2_{1,\ldots ,k-1}, \end{aligned}$$

where $R^2_{1,\ldots ,k}$, defined in (1), refers to a multicomponent *FMM* model with $K=k$ components. For atypical patterns, the identification is done using thresholds which have been checked over many previous fits to a wide variety of ECG patterns in Physionet.

The initial values for the components to start the backfitting are those of the waves assigned so far and zero for the rest. The algorithm finishes when there is no significant increase in the percentage of variance explained or when a maximum number of iterations is attained. An increase of less than 0.01% in the percentage of variance explained and a maximum of 10 iterations has been used in the analysis of the QT database.

**M step**: The backfitting algorithm is designed by fitting a single *FMM* component succesively to the residuals. To fit a single component, an adapted algorithm from that in^29^ is developed. The numbers of backfitting passes depend on the initialization. In the first M step up to 5 full turns of the backfitting are made.

**I step**: The *R* is assigned in the first place. *R* wave corresponds to the component, in the top five, with highest *PV* between components close to $t^{QRS}$, $\pi /2<\beta <5\pi /3$ (with a crest not a trough), $\omega <0.12$ (sharp) and maximum $\mu (t_J^U)$ (exceptionally the second maximum). Next, preassignation of *P*, *Q*, *S* and *T* to the free components among the first five is done using $\alpha _P \le \alpha _Q \le \alpha _R\le \alpha _S \le \alpha _T$. This preassigment corresponds to the final assignment in typical patterns. Successive steps are needed when the preassignation components do not exhibit the expected wave morphology features, known from literature; it can be due to the absence of a wave or to the presence of noisy components. New assignations of letter to components are conducted using thresholds on the *FMM* parameters that represent the previous knowledge. For instance, thresholds to decide between *P* or *Q*, are derived assuming that *Q* is between *P* and *R* ($\alpha _P\le \alpha _Q \le \alpha _R$), *Q* is often sharper ($\omega _Q< \omega _P$), and *Q* has a trough, while *P* has a crest. Noisy components are detected with small *PV*’s and $\omega$ values.

The outputs will be considered satisfactory (OK) only when the five letters are assigned and the parameters of the corresponding components describe the expected morphology.

Figure 4 shows a flowchart of the algorithm where different colors are used for M and I steps. The R code to implement the algorithm is available from corresponding author on reasonable request.

### Statistical methods and validation measures

Next, we briefly describe the statistical methods conducted and the validation measures used in the paper.

On the one hand, Fisher LDA has been considered as the method to discriminate among patterns and patients, a basic reference for learning about LDA is^45^. It is applied together with the one-leave-out approach which gives an unbiased estimate of the error rate.

On the other hand, the coefficient of determination used to measure the global goodness of fit is given by:

1 $$\begin{aligned} R^2=1-\frac{\sum _{i=1}^n (X(t_i)-{\hat{\mu }}(t_i))^2}{\sum _{i=1}^n (X(t_i)-{\overline{X}})^2}. \end{aligned}$$

For the analysis of simulation results, several mean squared error (MSE) measurements are considered to quantify the goodness of fit. Besides, the coefficient of variation measures are defined for Euclidean and angular parameters to measure consistency and accuracy. All these measures are defined throughout the Supplementary Information.

The measures to assess the wave detection of fiducial marks are: sensitivity ($Se=\frac{TP}{TP+FN}$), positive predictive value ($PPV=\frac{TP}{TP+FP}$), detection error rate ($DER=\frac{FP+FN}{TP+FN}$) and *F*1 score ($F1=\frac{2TP}{2TP+FP+FN}$), where *TP* is the number of true positive detections, *FN* stands for the number of negative detections and *FP* stands for the number of false positive detections, that is, when the fiducial mark is outside the range of ±75ms from the annotated mark.

## Supplementary information

Supplementary material 1
