# Supplementary material for: The hidden waves in the ECG uncovered revealing a sound automated interpretation method
Source: Sci Rep. 2021 Feb 12;11:3724. doi: 10.1038/s41598-021-82520-w (PMC7881027; doi:10.1038/s41598-021-82520-w)
Supplement: Supplementary file 1 — Supplementary material 1 [file 41598_2021_82520_MOESM1_ESM.pdf]

Supplementary Information for “ The hidden waves in the ECG uncovered revealing a sound automated interpretation method”

Cristina Rueda[1,*], Yolanda Larriba[1], and Adrian Lamela[1]

[1]Department of Statistics and Operations Research, Universidad de Valladolid, Valladolid, Spain

1 Simulations results

1.1 Simulations Design

A segment of the ECG is reproduced by repeatedly running a data-generating model defined by a signal $\mu(t)$, plus a Gaussian noise term. A typical pattern and six common atypical patterns are considered for the simulations. The exact values for $\mu^p(t); p = 1, ..., 7$; comes from original signals in Physionet (smoothing the original observed data using splines, except for the QRS that is as observed). The typical pattern, $\mu^1(t)$, corresponds the data in Figure 1 in the main document, while the other six patterns are shown in Figure S1.

The patterns are labelled by their prominent features as: 1.-Typical (TYP); 2.-Prominent Positive T wave (PPT); 3.-ST elevation (STE); 4.-ST depression (STD); 5.-Wide and Inverted QRS (WIQ); 6.-Flat T Wave (FLT) and 7.-Inverted T Wave (INV). These patterns have been selected as they are symptoms of different pathologies. In particular, pattern WIQ is typical of a premature ventricular contraction, where the QRS complex occurs earlier than expected in the cardiac cycle, is not preceded by a P wave and has a large duration. Also, the T wave is large and occurs in the opposite direction of the QRS complex. Causes include myocardial ischemia, structural heart disease, and many other risk factors [1]. Pattern STE is mainly regarded as a sign of myocardial ischemia although ST elevation is also associated with other processes including pericarditis or hyperkalemia [2]. Pattern STD, has an opposite morphology to STE for wave T that is the depression of ST segment; a variety of causes explain this morpohology [3]. Finally, patterns PPT, FLT and INV correspond to different morphologies for the T wave associated to pathologies, such as the earliest-described electrocardiographic sign of

acute ischemia [4].

The model that generate the simulated data is:

$$X(t_i) = \mu^p(t_i) + e(t_i); i = 1, ..., n;\ p = 1, ..., 7,$$

where $e(t_i) \sim N(0, \sigma_i^2); i = 1, ..., n$. Two configuration for the variance of the noise term are considered, labelled REAL and HIGH respectively. The REAL noise values replicate the real scenario and are estimated from residuals of the FMM_{ecg} model fitted to the QT database. Specifically, those values are $\sigma_i^2 = 6.25 * 10^{-4}$ for the t_i within QRS complex and $\sigma_i^2 = 10^{-2}$ for the rest of the time points. The HIGH noise values are $\sigma_i^2 = 10^{-2}$ within the QRS and $9 * 10^{-2}$ for those values which are outside.

For each scenario, $N = 100$ repetitions of $n = 150$ equally time spaced observations are generated, and two models were fitted to the data: FMM_{ecg} and GAU_7.

1.2 MSE measures

Several mean squared error (MSE) measures are considered to validate the results :

$$MSE_0 = \frac{1}{n}\sum_{i=1}^{n}(\hat{\mu}_0(t_i) - \mu(t_i))^2$$
$$MSE_D = \frac{1}{100}\sum_{j=1}^{100}\frac{1}{n}\sum_{i=1}^{n}(\hat{\mu}_j(t_i) - X_j(t_i))^2$$
$$MSE_M = \frac{1}{100}\sum_{j=1}^{100}\frac{1}{n}\sum_{i=1}^{n}(\hat{\mu}_0(t_i) - \hat{\mu}_j(t_i))^2$$

where, $\hat{\mu}_0(t)$ denote the fitted value for the signal without noise, and for the j monte-carlo trial, ($X_j(t_i)$, $\hat{\mu}_j(t_i)$ and $\hat{\theta}_j$; $i = 1, ..., n, j = 1, .., 100$) are the generated observations, the fitted values to those observations and the model-parameter estimators, respectively.

MSE values are given in Table S1 for both models: GAU_7 and FMM_{ecg}. While the values for the FMM_{ecg} are very low across different patterns, the values for GAU_7 are very high in specific patterns, such as patterns 2 and 6. The low MSE_M values in the HIGH scenario proves the robustness of the FMM_{ecg} against noise.

1.3 CV for FMM_{ecg} and GAU_7

Considering $\hat{\mu}_0(t)$ as the target model, $\hat{\theta}_0$ would be the true parameter value. Coefficient of variation measures are defined for Euclidean parameters in (1) and (2) and for angular parameters in (3) and (4). CV_{ϑ} is a measure of consistency and $CV_{\hat{\vartheta}}$ is a measure of accuracy. In the case of angular parameters, we define $\vartheta_1 = \hat{\vartheta}_0 - \pi$ and $\vartheta_2 = \bar{\hat{\vartheta}} - \pi$. This

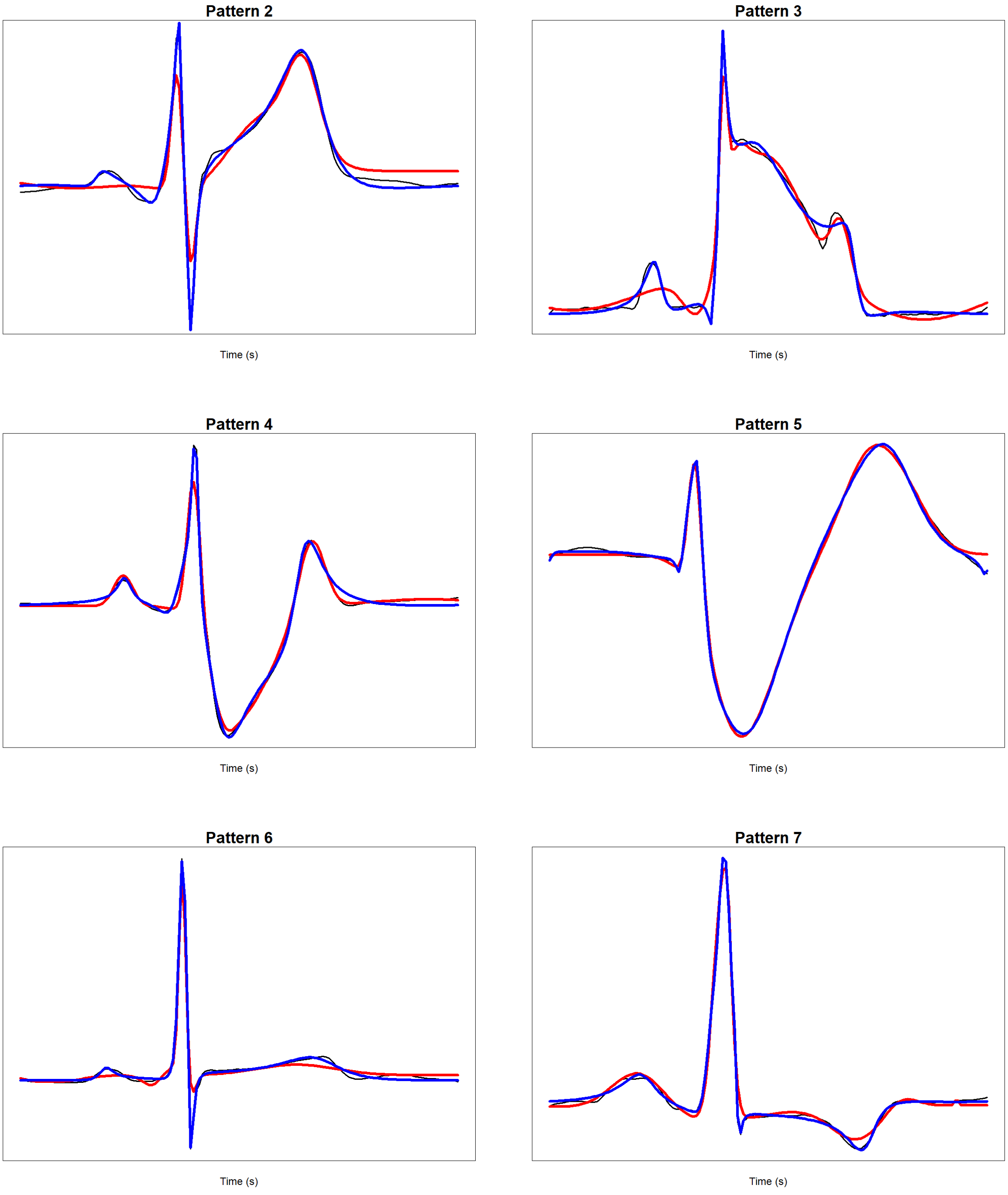

Figure S1: The six atypical patterns considered in simulations: $\mu()$ (black lines); FMM_{ecg} fit (blue lines) and GAU_7 fit (red lines). The blue line overlaps the black line when it is hidden

Table S1: MSE results from simulations.

	MSE_0		MSE_D		MSE_M	
Pattern Label	FMM_{ecg}	GAU_7	FMM_{ecg}	GAU_7	FMM_{ecg}	GAU_7
	REAL NOISE					
1-TYP	.010	.024	.019	.044	.011	.036
2-PPT	.007	.097	.016	.105	.008	.097
3-STE	.006	.030	.013	.036	.008	.030
4-STD	.008	.021	.015	.022	.009	.017
5-WIQ	.001	.003	.008	.008	.003	.003
6-FLT	.007	.068	.016	.068	.010	.060
7-INV	.004	.009	.012	.025	.006	.018
	HIGH NOISE					
1-TYP	.010	.035	.089	.113	.020	.044
2-PPT	.007	.111	.083	.170	.021	.103
3-STE	.006	.027	.064	.085	.015	.035
4-STD	.008	.020	.067	.077	.020	.021
5-WIQ	.001	.002	.062	.062	.020	.008
6-FLT	.007	.051	.083	.138	.018	.067
7-INV	.004	.015	.080	.087	.022	.024

translation around π is to avoid problems with zero values.

$$CV_{\vartheta} = \sqrt{\frac{\frac{1}{N}\sum_{j=1}^{N}(\hat{\vartheta}_0 - \hat{\vartheta}_j)^2}{\hat{\vartheta}_0^2}} \tag{1}$$

$$CV_{\hat{\vartheta}} = \sqrt{\frac{\frac{1}{N}\sum_{j=1}^{N}(\hat{\vartheta}_j - \bar{\hat{\vartheta}})^2}{\bar{\hat{\vartheta}}^2}} \tag{2}$$

$$CV_{\vartheta} = \sqrt{\frac{\frac{1}{N}\sum_{j=1}^{N}((\hat{\vartheta}_j - \vartheta_1) - \pi))^2}{\pi^2}} \tag{3}$$

$$CV_{\hat{\vartheta}} = \sqrt{\frac{\frac{1}{N}\sum_{j=1}^{N}((\hat{\vartheta}_j - \vartheta_2) - \pi)^2}{\pi^2}}, \tag{4}$$

Table S2 gives CV_{ϑ} and $CV_{\hat{\vartheta}}$ values for the 21 FMM_{ecg} and the 22 GAU_7 parameters. These values are much lower for FMM_{ecg} parameters across different patterns.

Furthermore, Figure S2 shows the distribution of $\hat{A}$'s, across simulation trials for both models. We have selected these parameters as illustration since they measure similar waveform aspects in both models. CV values and boxplots show that the FMM_{ecg} approach is very superior to the GAU_7 approach concerning consistency and accuracy.

1.4 P and T wave annotations.

The potential of the FMM_{ecg} parameters to detect the peaks of T and P waves is also confirmed with simulated data and performance measures are given in Table S3 for both scenarios (REAL and HIGH noise). Specifically, the DER values are 2.17% (21.67%) and 2.14% (3.71%) for P and T REAL noise (HIGH noise), respectively. Most of the detection errors happen in pattern 2, while for patterns 1, 3, 5 and 7 the detection error rate is almost null. No comparisons with GAU_7 are provided because the wave detection question is not solved using GAU_7.

Compared with the analysis of QT database results, given in the main document, note that the REAL noise results are better here; this is because the number of FP is reduced drastically in simulations as the true fiducial point is well-known, hence the causes behind many of the FP registered in the QT database analysis, do not occur in simulations. On the other hand, the results for the HIGH noise scenario are worse for the P wave, as expected, because is more affected by noise, as it is generally less prominent than the T wave.

Table S2: CV_{ϑ} and $CV_{\hat{\vartheta}}$ values for the 21 FMM_{ecg} and the 21 GAU_7 parameters

Model	CV_{ϑ}							$CV_{\hat{\vartheta}}$						
FMM	TYP	PPT	STE	STD	WIQ	FLT	INV	TYP	PPT	STE	STD	WIQ	FLT	INV
M	0.061	0.688	0.058	0.087	0.374	0.329	0.206	0.027	0.899	0.045	0.086	0.239	0.185	0.159
A_P	0.069	0.141	0.096	0.163	NA	0.237	0.129	0.064	0.141	0.059	0.144	NA	0.159	0.130
A_Q	0.073	0.102	0.125	0.117	0.505	0.205	0.104	0.034	0.088	0.088	0.103	0.374	0.142	0.063
A_R	0.058	0.051	0.014	0.027	0.144	0.167	0.091	0.013	0.039	0.014	0.013	0.116	0.110	0.034
A_S	0.025	0.098	0.057	0.019	0.256	0.086	0.089	0.018	0.076	0.035	0.019	0.260	0.042	0.060
A_T	0.018	0.019	0.020	0.021	0.186	0.063	0.047	0.017	0.019	0.019	0.021	0.183	0.049	0.047
w_P	0.076	0.495	0.518	0.224	NA	0.358	0.154	0.072	0.497	0.317	0.230	NA	0.348	0.119
w_Q	0.099	0.142	0.280	0.192	0.564	0.104	0.388	0.052	0.143	0.231	0.195	0.551	0.100	0.290
w_R	0.039	0.096	0.120	0.025	0.227	0.049	0.091	0.013	0.049	0.071	0.022	0.207	0.034	0.057
w_S	0.126	0.179	0.026	0.041	0.229	0.536	0.169	0.034	0.134	0.020	0.029	0.226	0.525	0.086
w_T	0.034	0.044	0.034	0.028	0.112	1.067	0.076	0.031	0.041	0.034	0.027	0.106	0.597	0.074
α_P	0.009	0.085	0.014	0.051	NA	0.056	0.049	0.007	0.083	0.011	0.051	NA	0.045	0.049
α_Q	0.001	0.014	0.004	0.021	0.008	0.003	0.016	0.001	0.011	0.004	0.020	0.008	0.002	0.012
α_R	0.001	0.003	0.001	0.000	0.004	0.004	0.002	0.001	0.003	0.000	0.000	0.004	0.002	0.001
α_S	0.000	0.002	0.023	0.003	0.378	0.002	0.004	0.000	0.002	0.012	0.003	0.383	0.002	0.002
α_T	0.004	0.007	0.005	0.003	0.096	0.354	0.013	0.004	0.006	0.004	0.003	0.089	0.362	0.011
β_P	0.056	0.339	0.051	0.308	NA	0.331	0.172	0.045	0.339	0.043	0.307	NA	0.257	0.172
β_Q	0.017	0.104	0.104	0.173	0.800	0.038	0.196	0.017	0.090	0.082	0.173	0.837	0.034	0.135
β_R	0.010	0.073	0.027	0.007	0.050	0.114	0.030	0.009	0.067	0.015	0.007	0.049	0.053	0.010
β_S	0.006	0.042	0.035	0.016	1.800	0.144	0.080	0.006	0.041	0.020	0.014	0.599	0.145	0.056
β_T	0.011	0.023	0.033	0.012	0.219	0.142	0.048	0.011	0.019	0.024	0.012	0.199	0.134	0.039
Gauss	TYP	PPT	STE	STD	WIQ	FLT	INV	TYP	PPT	STE	STD	WIQ	FLT	INV
M	0.522	0.514	0.752	0.206	1.414	0.846	1.054	0.533	0.386	1.133	0.190	44.446	0.468	0.583
A_1	0.802	0.915	1.434	5.346	2.463	10.429	3.165	1.341	1.424	1.224	1.437	2.509	1.952	1.232
A_2	4.124	3.700	1.006	1.101	1.832	3.481	6.639	2.070	3.952	3.500	2.641	21.871	2.001	2.699
A_3	4.515	1.098	1.581	1.515	2.051	0.897	2.983	1.737	2.656	3.422	8.652	3.853	2.032	1.760
A_4	1.540	3.040	1.974	1.427	2.119	6.516	0.951	2.256	1.813	3.219	6.205	3.900	1.552	2.851
A_5	4.416	1.957	2.652	1.478	14.300	11.416	1.239	2.350	3.459	3.417	20.132	68.260	2.246	2.880
A_6	1.107	4.502	1.390	1.297	0.989	21.039	2.468	1.701	2.244	4.889	2.131	2.793	1.898	2.328
A_7	4.873	1.617	1.116	2.034	1.156	1.348	2.065	1.898	10.597	7.613	3.667	9.937	2.013	2.453
S_1	0.573	0.404	1.037	1.198	0.335	0.533	0.427	0.467	0.414	0.365	0.363	0.343	0.457	0.446
S_2	4.338	2.675	0.532	0.407	0.369	0.562	0.400	0.476	0.378	0.531	0.272	0.381	0.457	0.422
S_3	0.518	0.673	0.577	0.267	0.470	0.568	0.500	0.467	0.497	0.486	0.277	0.458	0.431	0.418
S_4	0.499	0.540	1.123	0.335	0.431	0.573	0.487	0.520	0.441	0.535	0.278	0.397	0.463	0.368
S_4	0.986	0.419	0.540	0.314	0.498	3.863	0.508	0.508	0.461	0.543	0.296	0.364	0.450	0.416
S_6	0.559	0.534	0.507	0.384	0.739	0.410	0.423	0.463	0.459	0.585	0.316	0.390	0.441	0.387
S_7	0.462	0.535	0.651	0.279	0.448	0.525	1.275	0.485	0.457	0.467	0.281	0.400	0.432	0.408
W_1	3.408	0.583	0.601	0.683	0.485	34.829	3.629	0.677	0.713	0.751	0.674	0.521	1.211	0.769
W_2	0.605	0.657	0.633	4.078	0.414	3.227	1.096	0.758	0.771	0.653	0.760	0.447	1.356	0.784
W_3	17.161	6.002	2.845	0.574	0.506	7.138	0.712	0.854	0.788	0.705	0.675	0.478	1.181	0.830
W_4	1.042	5.249	0.604	1.060	0.447	74.258	3.114	0.750	0.812	0.681	0.643	0.461	1.198	0.814
W_5	2.186	0.586	0.658	1.632	45.375	0.772	1.464	0.664	0.720	0.793	0.708	0.422	1.113	0.805
W_6	5.199	0.635	3.189	1.444	4.393	0.766	1.129	0.804	0.807	0.606	0.723	0.481	1.070	0.849
W_7	0.615	5.679	8.528	0.593	0.408	18.138	0.673	0.770	0.832	0.692	0.735	0.421	1.032	0.841

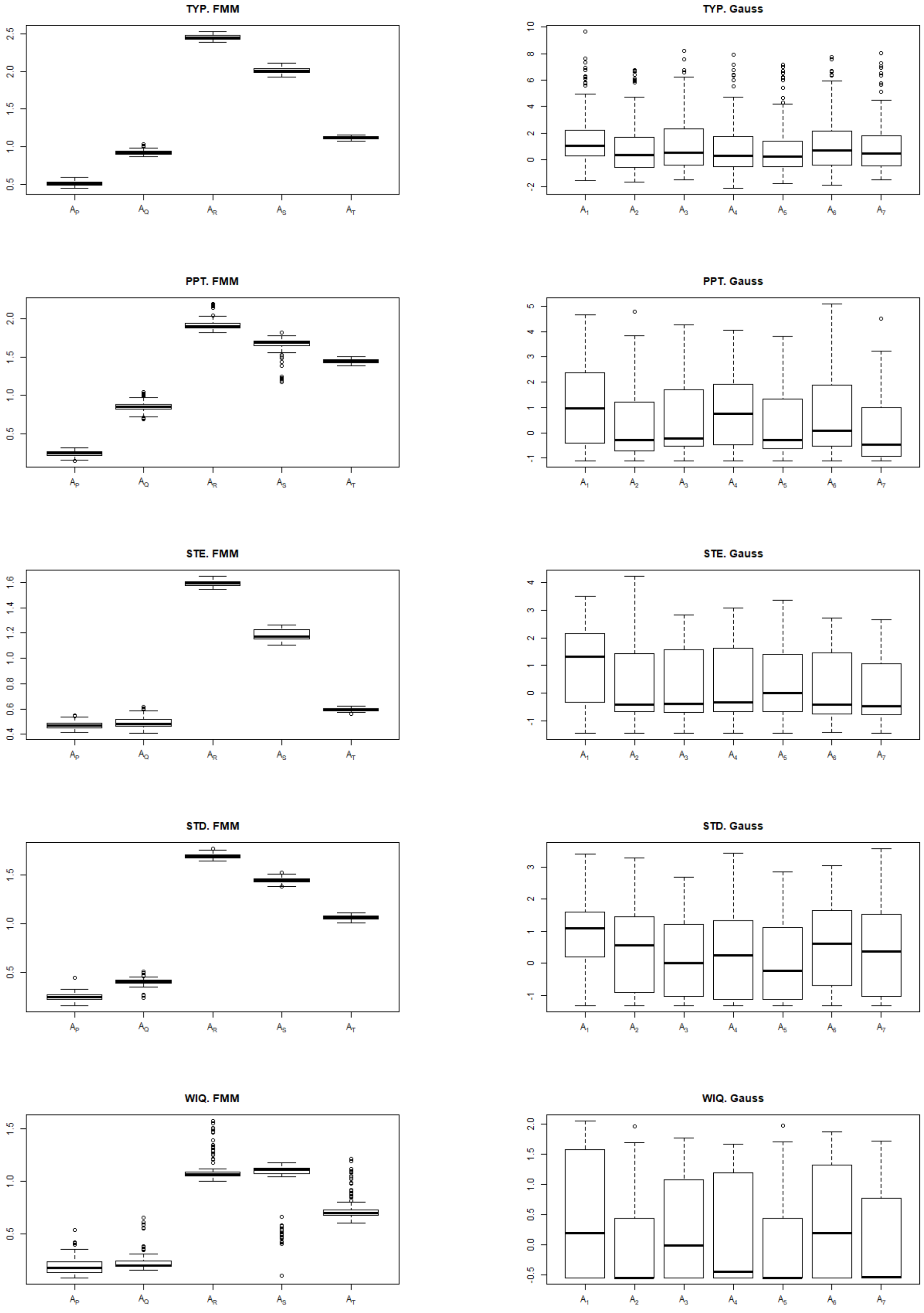

Figure S2: Box plots for $\hat{A}$'s from simulations. FMM_{ecg} parameters (left panels) and GAU_7 parameters (right panels) for different patterns

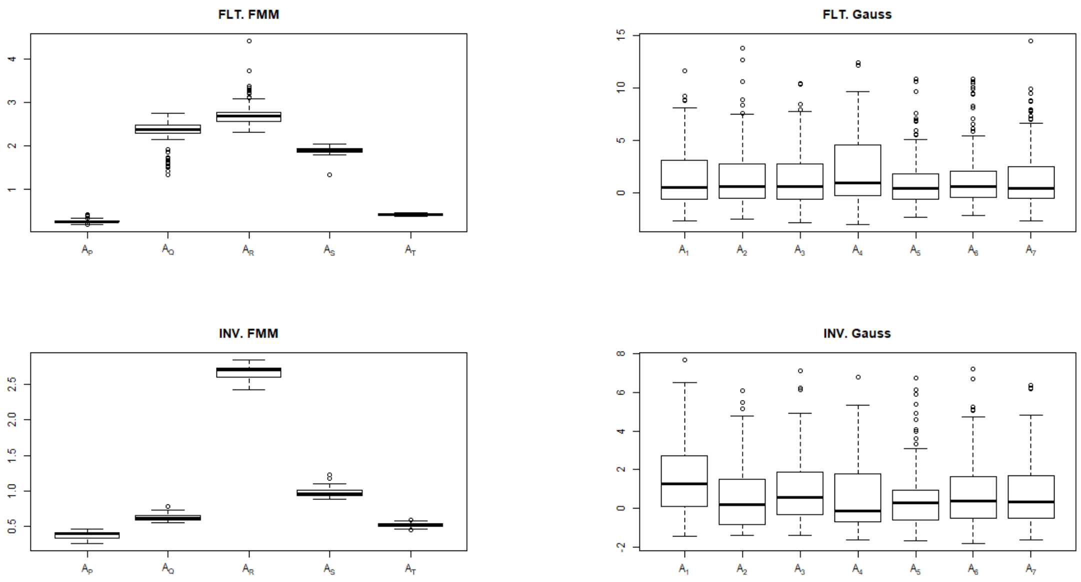

Figure S2: Box plots for $\hat{A}$'s from simulations. FMM_{ecg} parameters (left panels) and GAU_7 parameters (right panels) for different patterns

Table S3: Summary performance measures for P and T wave detection in simulated data.

P Wave

Noise	No. beats	TP	FP	FN	Se(%)	PPV(%)	DER(%)	$F1$(%)
REAL	600	597	10	3	99.50	98.35	2.17	98.92
HIGH	600	554	84	46	92.33	86.83	21.67	89.5

T Wave

Noise	No. beats	TP	FP	FN	Se(%)	PPV(%)	DER(%)	$F1$(%)
REAL	700	700	15	0	100	97.90	2.14	98.94
HIGH	700	700	26	0	100	96.42	3.71	98.18

1.4.1 Discrimination analysis

The power of the FMM_{ecg} and GAU_7 parameters to discriminate the seven patterns have been also analysed using the techniques Fisher Linear Discriminant Analysis (LDA) and K-nearest neighbour and the one-leave-out rule to estimate the error rate. Firstly, we analayse the REAL noisy scenario. Only five parameters from $FMM_{ecg} : \omega_Q, \omega_R, \omega_S, \beta_R, t_R^U$; are needed to correctly classify the 700 hearbeats, that is, the error rate is 0% for FMM_{ecg} while an error rate is 55% using the GAU_7 parameters; similar results were obtained with the k-nearest neighbours approach. Also similar conclusions arise with the analysis of HIGH noise data. Moreover, compared with others alternatives methods as [5] or [6], the results using FMM_{ecg} parameters are also quite good.

To illustrate graphically the potential of FMM_{ecg} approach for the classification task we have, for the REAL noisy scenario, the plot of the two first principal components calculated from the five selected FMM_{ecg} parameters. This plot is displayed in Figure S3 (a) versus the plot of the two first principal components calculated from the 21 main GAU_7 parameters, displayed in Figure S3 (b). The percentage of the variance that is explained by the first two principal components are 72% and 17%, respectively.

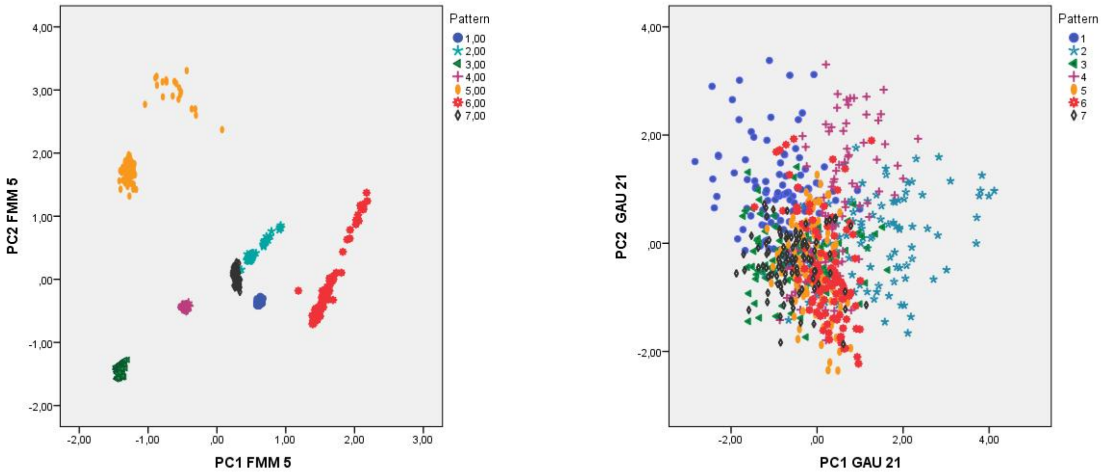

(a) (b)

Figure S3: First against second principal components from the analysis (a) FMM_{ecg} parameters and (b) GAU_7 coefficients. Colors represent different patterns.

2 QT Database Results. Extension

This section reinforces QT database analysis given in the main text including parameter estimation, fiducial mark detection and classification issues for the 105 patients in the database.

2.1 FMM_{ecg} estimates. Fiducial marks

Next, patient specific results from FMM_{ecg} are given. On the one hand, Figures S4 to S10 show the observed and FMM_{ecg} fitted values for representative beats for each of the 105 QT patients.

On the other hand, individual statistics for the main parameters estimators are given in Table S4. Tables S5 and S6 give measures to assess the detection of P and T waves. Finally, Figures S11 to S16 show cases where the FMM_{ecg} correctly locates the wave but the annotation is different to Physionet annotation. Specifically:

- Figure S11: T wave FMM_{ecg} annotation is right in view of the first signal displayed (Physionet T wave annotation likely comes from the second signal).
- Figures S12: T wave FMM_{ecg} annotation is located at the maximum while Physionet annotation is at the minimum.
- Figures S13: T wave FMM_{ecg} annotation is located at the minimum and Physionet annotation is at the maximum.
- Figure S14: No P wave FMM_{ecg} annotation. Physionet P annotation is probably due to the fact that P is present in other beats.
- Figure S15: No P wave FMM_{ecg} annotation. Physionet P annotation is probably due to the fact that P is prominent in the second signal.
- Figure S16: P wave FMM_{ecg} annotation is located at the minimum while Physionet annotation is at the maximum.

Table S4: Patient specific statistics, median and percentile intervals for t^{FI}, ω and β estimators. t^{FI} are specifically detailed in Section 2.2.2 of the main document. $[P_5, P_{95}] = arc(P_5, P_{95})$, for the angular parameters β and t^{FI}. NA is registered when the corresponding wave is not assigned in at least the 10% of the beats. Patients are sorted alphabetically

		P Wave			Q Wave			R Wave			S Wave			T Wave		
	Patient	t^{FI}	ω	β	t^{FI}	ω	β	t^{FI}	ω	β	t^{FI}	ω	β	t^{FI}	ω	β
P_{50}	*sel0104*	1.588	0.062	3.166	2.426	0.023	0.848	2.415	0.038	2.790	2.440	0.022	5.457	3.649	0.296	3.519
P_5	*sel0104*	1.513	0.053	2.566	2.332	0.019	0.434	2.357	0.034	2.660	2.265	0.020	5.164	3.603	0.253	2.820
P_{95}	*sel0104*	1.619	0.094	3.596	2.503	0.026	1.683	2.476	0.042	2.901	2.483	0.025	5.918	3.713	0.329	3.977
P_{50}	*sel0106*	1.553	0.070	3.565	2.373	0.037	1.674	2.402	0.024	4.893	2.702	0.034	1.096	4.511	0.151	2.591
P_5	*sel0106*	1.514	0.062	3.149	2.335	0.035	1.589	2.371	0.022	4.794	2.641	0.030	0.558	4.410	0.128	2.162
P_{95}	*sel0106*	1.612	0.079	3.832	2.434	0.039	1.769	2.467	0.025	4.956	2.831	0.043	1.584	4.634	0.266	2.834
P_{50}	*sel0107*	1.764	0.059	3.047	2.414	0.025	1.553	2.492	0.040	3.687	2.060	0.027	6.140	4.186	0.155	2.638
P_5	*sel0107*	1.709	0.053	2.859	2.360	0.024	1.429	2.448	0.038	3.498	1.326	0.024	5.944	4.109	0.115	2.325
P_{95}	*sel0107*	1.802	0.072	3.374	2.451	0.027	1.808	2.531	0.044	3.825	4.110	0.033	0.127	4.252	0.239	3.078
P_{50}	*sel0110*	1.131	0.055	5.357	2.249	0.027	2.144	2.292	0.027	3.301	2.836	0.042	0.045	4.124	0.176	3.552
P_5	*sel0110*	6.066	0.019	3.832	2.069	0.015	0.948	2.211	0.015	2.699	0.568	0.026	5.494	4.055	0.155	3.254
P_{95}	*sel0110*	1.296	0.206	0.054	2.341	0.057	2.443	2.334	0.034	4.195	4.653	0.047	0.405	4.331	0.216	3.647
P_{50}	*sel0111*	1.440	0.095	3.407	2.414	0.021	0.569	2.427	0.045	2.805	2.529	0.014	4.702	4.172	0.206	2.971
P_5	*sel0111*	1.384	0.085	3.106	2.135	0.019	6.281	2.390	0.043	2.668	2.491	0.013	4.625	4.101	0.194	2.871
P_{95}	*sel0111*	1.488	0.114	3.716	4.475	0.024	1.503	2.455	0.049	2.928	2.553	0.015	4.805	4.218	0.218	3.079
P_{50}	*sel0112*	1.779	0.163	1.923	2.417	0.024	1.592	2.471	0.041	3.461	2.499	0.015	5.596	3.817	0.176	3.256
P_5	*sel0112*	5.520	0.051	5.637	2.330	0.019	1.141	2.414	0.021	3.053	2.164	0.009	5.190	3.778	0.164	3.061
P_{95}	*sel0112*	3.101	0.364	3.427	2.458	0.035	2.103	2.514	0.051	4.387	2.806	0.018	0.228	3.966	0.191	3.445

		P Wave			Q Wave			R Wave			S Wave			*T* Wave		
	Patient	t^{FI}	ω	β	t^{FI}	ω	β	t^{FI}	ω	β	t^{FI}	ω	β	t^{FI}	ω	β
P_{50}	*sel0114*	1.927	0.100	2.686	2.514	0.022	0.644	2.543	0.031	2.642	2.575	0.014	5.322	3.675	0.142	3.419
P_5	*sel0114*	1.179	0.041	6.155	1.688	0.011	5.557	1.716	0.026	2.451	1.751	0.010	5.203	2.966	0.110	3.124
P_{95}	*sel0114*	3.893	0.371	3.983	3.372	0.057	2.074	2.929	0.039	3.109	2.962	0.019	5.493	4.404	0.202	3.691
P_{50}	*sel0116*	0.760	0.174	3.598	2.436	0.040	1.139	2.458	0.042	3.255	2.520	0.020	5.354	4.466	0.158	3.388
P_5	*sel0116*	0.299	0.015	2.451	2.345	0.019	0.601	2.422	0.035	3.134	2.454	0.014	5.152	4.339	0.137	3.158
P_{95}	*sel0116*	1.412	0.385	4.882	2.581	0.049	1.621	2.526	0.051	3.464	2.574	0.024	5.485	4.576	0.183	3.595
P_{50}	*sel0121*	1.118	0.165	4.007	2.347	0.034	2.109	2.426	0.044	3.324	2.369	0.033	5.701	4.683	0.189	3.335
P_5	*sel0121*	1.080	0.129	3.950	2.320	0.031	1.868	2.398	0.042	3.255	2.032	0.028	5.286	4.648	0.178	3.203
P_{95}	*sel0121*	1.158	0.199	4.102	2.384	0.036	2.243	2.460	0.046	3.527	2.448	0.036	6.101	4.742	0.204	3.480
P_{50}	*sel0122*	1.196	0.103	4.305	2.339	0.042	1.637	2.382	0.044	3.775	4.084	0.043	0.092	4.631	0.175	3.515
P_5	*sel0122*	1.157	0.090	4.167	2.311	0.040	1.547	2.352	0.042	3.677	3.085	0.040	6.277	4.577	0.166	3.445
P_{95}	*sel0122*	1.226	0.114	4.404	2.361	0.045	1.929	2.403	0.046	4.123	5.852	0.047	0.313	4.713	0.185	3.626
P_{50}	*sel0124*	1.186	0.119	4.082	2.403	0.046	1.653	2.445	0.038	3.670	2.442	0.024	5.930	4.548	0.291	3.618
P_5	*sel0124*	1.091	0.096	3.905	2.321	0.039	1.505	2.401	0.028	3.478	2.191	0.016	5.609	4.418	0.248	3.504
P_{95}	*sel0124*	1.279	0.152	4.276	2.452	0.050	2.029	2.495	0.042	4.250	3.355	0.029	0.272	4.672	0.428	3.760
P_{50}	*sel0126*	1.081	0.114	1.926	1.083	0.048	5.863	2.326	0.078	2.006	2.156	0.032	5.867	3.870	0.170	2.750
P_5	*sel0126*	0.976	0.063	1.426	0.934	0.019	5.626	2.269	0.067	1.859	2.064	0.027	5.786	3.797	0.081	2.417
P_{95}	*sel0126*	1.147	0.147	2.889	2.124	0.066	1.527	2.372	0.087	2.132	2.245	0.034	5.937	3.920	0.242	3.575
P_{50}	*sel0129*	1.449	0.100	3.280	2.426	0.029	2.641	2.445	0.022	4.449	2.781	0.053	1.248	3.928	0.159	3.598
P_5	*sel0129*	5.271	0.034	2.175	2.334	0.018	1.153	2.218	0.020	3.172	2.046	0.023	5.903	3.764	0.143	3.485
P_{95}	*sel0129*	1.741	0.277	5.541	2.660	0.130	3.088	2.533	0.041	4.722	3.266	0.152	1.626	4.320	0.183	3.848
P_{50}	*sel0133*	1.386	0.066	4.979	2.362	0.048	1.752	2.402	0.036	4.485	3.044	0.034	0.284	4.127	0.186	3.726
P_5	*sel0133*	1.343	0.057	4.645	2.305	0.043	1.561	2.357	0.033	4.422	2.880	0.032	0.161	4.019	0.178	3.662
P_{95}	*sel0133*	1.460	0.079	5.494	2.421	0.058	1.832	2.457	0.038	4.573	3.467	0.035	0.380	4.203	0.193	3.778
P_{50}	*sel0136*	1.632	0.055	3.153	2.419	0.026	1.299	2.431	0.032	3.226	2.434	0.028	5.452	4.240	0.128	2.682
P_5	*sel0136*	1.310	0.044	2.241	2.060	0.021	0.609	2.372	0.027	3.101	2.219	0.018	5.030	4.101	0.093	2.095
P_{95}	*sel0136*	1.793	0.124	4.307	2.575	0.040	1.605	2.576	0.049	3.434	2.521	0.042	5.791	4.933	0.199	3.185
P_{50}	*sel0166*	1.620	0.082	3.170	2.373	0.024	0.954	2.408	0.036	3.183	2.428	0.018	5.151	4.289	0.189	2.624
P_5	*sel0166*	1.540	0.034	2.426	2.327	0.021	0.569	2.357	0.032	2.884	2.386	0.014	4.933	4.209	0.141	2.241
P_{95}	*sel0166*	1.728	0.228	4.038	2.469	0.027	1.254	2.501	0.040	3.402	2.528	0.023	5.466	4.430	0.285	3.121
P_{50}	*sel0170*	1.636	0.060	2.699	2.442	0.038	1.349	2.452	0.034	3.129	2.468	0.025	5.269	4.110	0.145	3.354
P_5	*sel0170*	1.593	0.054	2.421	2.402	0.036	1.283	2.414	0.033	3.091	2.423	0.024	5.206	4.046	0.139	3.326
P_{95}	*sel0170*	1.680	0.067	2.947	2.493	0.041	1.389	2.498	0.035	3.190	2.515	0.026	5.335	4.186	0.148	3.397
P_{50}	*sel0203*	0.823	0.224	3.363	2.379	0.046	1.276	2.452	0.075	3.451	2.668	0.034	2.753	5.068	0.260	1.379
P_5	*sel0203*	0.577	0.159	2.995	2.189	0.038	1.109	2.254	0.066	3.405	2.443	0.019	2.106	4.645	0.228	1.158
P_{95}	*sel0203*	1.097	0.279	3.545	2.493	0.049	1.418	2.560	0.086	3.514	2.782	0.040	3.397	5.309	0.297	1.464
P_{50}	*sel0210*	1.278	0.152	4.160	2.408	0.042	1.048	2.439	0.047	3.171	2.516	0.028	4.824	4.442	0.166	3.248
P_5	*sel0210*	1.143	0.088	3.776	2.259	0.029	0.045	2.355	0.043	2.884	2.419	0.019	4.536	4.311	0.151	3.131
P_{95}	*sel0210*	1.434	0.260	4.497	2.593	0.050	1.694	2.505	0.053	3.425	2.573	0.035	5.579	4.573	0.178	3.312
P_{50}	*sel0211*	0.607	0.273	2.817	2.264	0.047	1.456	2.455	0.078	3.605	2.507	0.036	4.818	4.719	0.276	3.142
P_5	*sel0211*	0.547	0.231	2.568	2.247	0.044	1.237	2.431	0.058	3.269	2.469	0.028	4.486	4.692	0.254	3.019
P_{95}	*sel0211*	0.662	0.301	3.025	2.289	0.050	1.593	2.480	0.083	4.048	2.536	0.043	5.042	4.749	0.297	3.293
P_{50}	*sel0303*	1.399	0.126	3.902	2.354	0.049	1.719	2.469	0.053	3.946	2.832	0.036	1.321	4.646	0.127	2.030
P_5	*sel0303*	1.378	0.099	3.590	2.334	0.046	1.507	2.450	0.047	3.897	2.786	0.019	0.935	4.608	0.120	1.942
P_{95}	*sel0303*	1.423	0.154	4.037	2.379	0.050	1.954	2.492	0.055	4.008	2.864	0.050	1.642	4.667	0.132	2.146
P_{50}	*sel0405*	1.245	0.175	4.459	2.300	0.026	1.496	2.397	0.079	2.746	2.522	0.028	4.962	4.567	0.209	3.637
P_5	*sel0405*	1.103	0.121	3.776	2.238	0.019	0.574	2.343	0.068	2.439	2.476	0.026	4.904	4.466	0.169	3.368
P_{95}	*sel0405*	1.408	0.277	4.851	2.494	0.037	2.006	2.452	0.084	2.881	2.569	0.032	5.156	4.635	0.248	3.910
P_{50}	*sel0406*	1.533	0.056	3.395	2.456	0.075	1.087	2.440	0.030	3.921	2.360	0.024	6.118	4.305	0.157	3.286
P_5	*sel0406*	5.525	0.031	1.757	2.354	0.042	0.762	2.406	0.017	3.726	1.054	0.018	5.621	4.246	0.132	3.025
P_{95}	*sel0406*	1.640	0.312	4.961	2.707	0.120	1.789	2.492	0.035	4.193	2.962	0.030	0.559	4.393	0.198	3.595
P_{50}	*sel0409*	0.738	0.120	3.627	2.380	0.070	1.085	2.406	0.092	4.411	2.819	0.036	0.819	5.430	0.264	1.452
P_5	*sel0409*	0.678	0.110	3.206	2.342	0.067	1.032	2.383	0.088	4.252	2.764	0.033	0.602	5.363	0.255	1.259
P_{95}	*sel0409*	0.791	0.197	3.816	2.472	0.093	1.150	2.450	0.099	4.479	2.908	0.050	1.103	5.550	0.316	1.501
P_{50}	*sel0411*	1.367	0.061	4.359	2.404	0.046	1.428	2.448	0.043	3.345	2.382	0.041	5.550	4.389	0.198	3.541
P_5	*sel0411*	1.217	0.041	3.414	2.354	0.041	0.705	2.391	0.040	3.143	2.059	0.038	5.291	4.307	0.188	3.371
P_{95}	*sel0411*	1.465	0.160	4.702	3.431	0.118	1.812	2.500	0.048	3.624	2.479	0.049	6.007	4.491	0.213	3.690
P_{50}	*sel0509*	1.232	0.128	5.423	2.429	0.037	1.268	2.484	0.035	3.638	2.500	0.025	5.281	4.322	0.204	3.385
P_5	*sel0509*	0.614	0.073	4.614	2.364	0.030	1.156	2.441	0.030	3.532	2.443	0.014	4.930	4.203	0.186	3.196
P_{95}	*sel0509*	1.496	0.203	5.844	2.478	0.047	1.488	2.521	0.037	3.807	2.555	0.028	5.387	4.416	0.225	3.568
P_{50}	*sel0603*	1.575	0.074	2.370	2.420	0.047	1.660	2.445	0.031	4.469	2.009	0.034	6.136	4.001	0.096	3.895
P_5	*sel0603*	1.491	0.052	1.826	1.972	0.036	6.151	2.384	0.028	1.862	0.352	0.027	5.317	3.914	0.085	3.723
P_{95}	*sel0603*	1.650	0.123	3.248	2.468	0.050	1.911	2.489	0.046	4.694	4.622	0.037	0.107	4.055	0.108	4.085
P_{50}	*sel0604*	1.443	0.167	3.560	2.541	0.043	1.817	2.582	0.051	3.418	2.691	0.113	3.594	4.824	0.270	2.549
P_5	*sel0604*	1.373	0.088	3.110	2.495	0.035	0.241	2.549	0.039	3.193	1.759	0.006	1.203	4.738	0.189	1.838
P_{95}	*sel0604*	1.519	0.200	4.224	3.130	0.049	2.105	2.617	0.055	3.599	2.806	0.230	5.786	4.857	0.323	3.293
P_{50}	*sel0606*	1.103	0.160	4.378	2.330	0.032	1.604	2.539	0.051	2.495	2.642	0.064	5.594	4.707	0.263	3.684
P_5	*sel0606*	0.994	0.137	4.094	2.265	0.014	0.327	2.494	0.031	2.158	2.439	0.041	4.900	4.637	0.205	3.483
P_{95}	*sel0606*	1.169	0.208	5.311	2.650	0.101	2.283	2.629	0.075	4.922	3.883	0.073	0.739	4.767	0.293	3.964
P_{50}	*sel0607*	1.128	0.131	4.054	2.363	0.038	1.685	2.440	0.049	3.401	2.464	0.024	5.475	5.210	0.144	1.798
P_5	*sel0607*	1.095	0.115	3.918	2.337	0.035	1.507	2.418	0.046	3.293	2.270	0.017	4.922	5.107	0.123	1.628
P_{95}	*sel0607*	1.190	0.146	4.264	2.395	0.041	1.807	2.475	0.053	3.465	2.909	0.031	6.229	5.272	0.165	2.029
P_{50}	*sel0609*	1.410	0.149	3.645	2.302	0.039	1.430	2.466	0.066	3.673	NA	NA	NA	4.549	0.142	2.436
P_5	*sel0609*	1.336	0.100	3.259	2.272	0.034	1.087	2.437	0.062	3.593	NA	NA	NA	4.445	0.112	2.005
P_{95}	*sel0609*	1.459	0.269	3.982	2.348	0.043	1.566	2.515	0.071	3.753	NA	NA	NA	4.611	0.167	2.893
P_{50}	*sel0612*	1.274	0.214	3.722	2.407	0.023	1.472	2.456	0.036	3.187	2.484	0.031	4.153	4.118	0.130	3.202
P_5	*sel0612*	1.192	0.060	2.727	1.972	0.014	6.033	2.432	0.032	2.971	1.457	0.025	3.952	4.055	0.121	2.343
P_{95}	*sel0612*	1.323	0.394	4.209	3.612	0.027	2.067	2.488	0.039	3.277	2.528	0.223	5.716	4.199	0.192	3.469
P_{50}	*sel0704*	1.402	0.077	3.866	2.282	0.034	1.604	2.353	0.058	4.601	2.296	0.093	5.613	4.330	0.161	3.548
P_5	*sel0704*	1.288	0.045	2.685	2.178	0.006	1.211	2.269	0.024	1.753	6.057	0.047	5.071	4.182	0.134	2.914

		P Wave			Q Wave			R Wave			S Wave			T Wave		
	Patient	t^{FI}	ω	β	t^{FI}	ω	β	t^{FI}	ω	β	t^{FI}	ω	β	t^{FI}	ω	β
P_{95}	*sel0704*	1.496	0.127	4.391	2.325	0.041	1.923	2.437	0.068	4.931	4.855	0.141	0.716	4.427	0.408	4.068
P_{50}	*sel100*	1.188	0.136	3.381	2.488	0.037	0.647	2.413	0.034	3.343	2.472	0.016	5.115	5.119	0.171	1.834
P_5	*sel100*	1.062	0.112	2.876	2.379	0.032	0.419	2.335	0.029	3.177	2.349	0.012	4.685	4.986	0.127	1.384
P_{95}	*sel100*	1.329	0.192	3.686	2.663	0.045	1.037	2.586	0.044	3.433	2.634	0.025	5.635	5.393	0.219	2.248
P_{50}	*sel102*	0.131	0.154	4.892	1.958	0.028	1.015	1.972	0.023	2.585	2.355	0.072	4.258	4.639	0.181	2.439
P_5	*sel102*	4.959	0.042	1.954	1.305	0.006	5.874	1.572	0.016	2.359	2.008	0.048	3.769	4.314	0.125	1.878
P_{95}	*sel102*	2.258	0.339	1.624	3.004	0.255	2.711	2.175	0.042	2.788	2.613	0.114	5.088	4.955	0.286	3.301
P_{50}	*sel103*	1.213	0.175	3.979	2.373	0.027	1.496	2.437	0.036	3.093	2.459	0.023	5.286	4.358	0.179	2.599
P_5	*sel103*	1.099	0.102	3.058	2.289	0.022	1.082	2.344	0.033	3.018	2.362	0.017	4.940	4.214	0.170	2.364
P_{95}	*sel103*	1.325	0.377	4.520	2.495	0.033	1.711	2.558	0.039	3.188	2.571	0.028	5.669	4.436	0.201	2.820
P_{50}	*sel104*	1.074	0.112	3.075	2.276	0.090	2.054	2.278	0.018	2.756	1.983	0.123	6.191	5.228	0.329	3.985
P_5	*sel104*	0.195	0.045	1.554	1.995	0.009	0.604	1.930	0.012	2.463	5.522	0.015	3.630	4.698	0.184	1.195
P_{95}	*sel104*	1.793	0.241	5.057	2.903	0.238	3.033	2.534	0.068	3.833	3.216	0.144	2.131	6.268	0.542	0.394
P_{50}	*sel114*	1.264	0.179	4.218	2.372	0.044	1.880	2.497	0.045	3.505	2.734	0.020	0.459	4.513	0.144	1.969
P_5	*sel114*	0.656	0.062	2.102	2.193	0.030	0.996	2.328	0.038	3.417	2.265	0.006	4.705	4.306	0.105	1.515
P_{95}	*sel114*	1.476	0.330	4.856	2.492	0.056	2.485	2.573	0.052	3.608	3.183	0.040	1.699	4.655	0.190	2.713
P_{50}	*sel116*	1.359	0.127	3.986	2.384	0.045	1.708	2.463	0.048	3.284	2.411	0.021	5.891	4.305	0.194	3.128
P_5	*sel116*	1.189	0.073	2.472	2.295	0.038	1.224	2.379	0.039	3.139	1.871	0.015	5.193	4.218	0.165	2.724
P_{95}	*sel116*	1.470	0.293	4.581	2.420	0.049	2.087	2.499	0.054	3.430	2.589	0.025	6.239	4.443	0.294	3.636
P_{50}	*sel117*	1.625	0.059	3.002	2.469	0.047	1.193	2.451	0.028	3.949	2.432	0.025	6.140	4.046	0.145	3.450
P_5	*sel117*	1.512	0.049	1.961	2.360	0.026	0.573	2.351	0.023	3.437	0.684	0.017	5.706	3.971	0.132	3.185
P_{95}	*sel117*	1.661	0.102	4.129	2.999	0.118	2.116	2.498	0.031	4.652	3.076	0.039	1.043	4.131	0.172	3.847
P_{50}	*sel123*	1.530	0.063	3.829	2.454	0.032	1.936	2.484	0.024	3.774	2.887	0.019	0.028	4.024	0.129	2.984
P_5	*sel123*	1.199	0.047	3.191	2.333	0.024	1.597	2.344	0.020	3.596	0.908	0.011	5.960	3.818	0.109	2.691
P_{95}	*sel123*	1.692	0.097	4.344	2.564	0.042	2.058	2.605	0.033	3.947	5.331	0.026	0.260	4.490	0.140	3.228
P_{50}	*sel14046*	1.413	0.053	3.952	2.279	0.045	1.888	2.347	0.025	3.523	3.576	0.058	0.072	4.271	0.188	3.593
P_5	*sel14046*	5.849	0.041	2.291	2.203	0.011	0.393	2.288	0.018	3.198	1.102	0.043	6.104	4.164	0.172	3.338
P_{95}	*sel14046*	1.479	0.137	6.139	3.673	0.211	2.201	2.431	0.035	4.126	0.139	0.075	0.342	4.345	0.213	3.882
P_{50}	*sel14157*	0.703	0.211	1.975	2.523	0.069	0.817	2.331	0.062	3.414	2.397	0.056	6.243	5.132	0.153	0.832
P_5	*sel14157*	0.593	0.097	1.324	2.410	0.046	0.544	2.315	0.055	3.322	0.239	0.028	5.671	5.023	0.130	0.657
P_{95}	*sel14157*	0.902	0.386	2.858	2.758	0.091	0.978	2.383	0.073	3.679	4.112	0.068	0.623	5.291	0.161	0.977
P_{50}	*sel14172*	1.564	0.065	3.055	2.362	0.067	1.439	2.340	0.018	3.824	2.249	0.034	5.743	4.840	0.295	4.303
P_5	*sel14172*	1.475	0.050	2.388	1.177	0.022	6.146	2.281	0.009	1.746	1.536	0.024	5.462	3.755	0.132	1.665
P_{95}	*sel14172*	1.682	0.115	5.542	2.510	0.110	2.300	2.430	0.056	4.852	3.055	0.044	0.381	5.197	0.690	5.263
P_{50}	*sel15814*	0.866	0.108	2.489	2.143	0.014	2.289	2.175	0.055	4.505	3.877	0.260	0.845	4.428	0.253	4.387
P_5	*sel15814*	0.815	0.092	2.164	2.113	0.010	2.040	2.142	0.051	4.220	3.810	0.251	0.714	4.374	0.227	4.231
P_{95}	*sel15814*	0.922	0.143	3.014	2.163	0.019	2.343	2.215	0.062	4.646	3.994	0.272	0.918	4.480	0.279	4.620
P_{50}	*sel16265*	1.072	0.212	3.241	2.969	0.040	0.106	2.379	0.063	2.862	2.473	0.038	4.501	4.630	0.326	2.689
P_5	*sel16265*	0.973	0.147	2.838	0.498	0.036	6.114	2.306	0.060	2.779	2.393	0.034	4.400	4.499	0.221	2.189
P_{95}	*sel16265*	1.301	0.364	3.705	4.994	0.044	0.450	2.475	0.072	3.133	2.571	0.044	4.846	4.769	0.396	3.075
P_{50}	*sel16272*	1.838	0.058	3.533	2.428	0.028	2.039	2.463	0.021	3.826	3.137	0.026	0.181	4.197	0.153	3.541
P_5	*sel16272*	1.799	0.050	2.823	2.401	0.023	1.899	2.426	0.019	3.584	2.677	0.015	5.530	4.125	0.145	3.126
P_{95}	*sel16272*	1.888	0.068	3.903	2.473	0.034	2.241	2.506	0.025	4.095	5.285	0.030	0.382	4.264	0.196	3.679
P_{50}	*sel16273*	1.429	0.146	4.313	2.530	0.031	0.237	2.415	0.050	2.934	2.444	0.042	4.628	4.421	0.157	2.736
P_5	*sel16273*	1.263	0.034	3.028	0.526	0.021	6.142	2.340	0.042	2.784	2.363	0.038	4.366	4.322	0.126	2.381
P_{95}	*sel16273*	1.543	0.277	4.628	5.074	0.038	1.081	2.486	0.055	3.086	2.520	0.047	4.886	4.566	0.194	3.019
P_{50}	*sel16420*	1.175	0.098	3.786	2.439	0.035	1.956	2.463	0.025	3.721	2.808	0.027	0.134	4.365	0.277	3.471
P_5	*sel16420*	1.072	0.071	3.132	2.349	0.023	1.463	2.410	0.021	3.517	1.802	0.021	5.470	4.188	0.229	2.581
P_{95}	*sel16420*	1.268	0.153	3.990	2.514	0.049	2.435	2.554	0.030	4.058	3.926	0.039	0.793	4.504	0.695	3.853
P_{50}	*sel16483*	1.083	0.076	3.978	2.363	0.032	1.610	2.410	0.032	3.681	3.656	0.043	0.081	4.372	0.169	3.713
P_5	*sel16483*	1.003	0.061	3.397	2.324	0.029	1.119	2.378	0.028	3.492	1.250	0.036	6.147	4.286	0.161	3.629
P_{95}	*sel16483*	1.138	0.094	4.336	2.423	0.038	1.759	2.430	0.036	3.981	6.054	0.050	0.594	4.411	0.176	3.791
P_{50}	*sel16539*	1.208	0.050	3.303	2.442	0.028	1.245	2.469	0.028	3.487	2.241	0.028	5.969	4.592	0.152	3.471
P_5	*sel16539*	0.893	0.032	2.613	2.249	0.020	0.799	2.288	0.022	3.288	1.671	0.022	5.659	4.139	0.113	3.280
P_{95}	*sel16539*	1.754	0.072	3.610	2.629	0.035	1.493	2.646	0.035	3.698	2.451	0.037	6.242	4.865	0.197	3.615
P_{50}	*sel16773*	1.455	0.053	3.418	2.426	0.038	0.805	2.445	0.045	3.438	2.344	0.040	5.585	4.057	0.178	3.504
P_5	*sel16773*	1.302	0.033	1.974	2.307	0.028	0.539	2.361	0.035	3.062	2.096	0.032	5.298	3.871	0.161	3.071
P_{95}	*sel16773*	1.583	0.290	4.075	2.558	0.045	1.143	2.513	0.051	3.704	2.466	0.049	5.971	4.149	0.243	3.731
P_{50}	*sel16786*	1.206	0.069	3.633	2.769	0.030	0.223	2.442	0.044	3.086	2.491	0.022	4.469	4.332	0.138	3.370
P_5	*sel16786*	1.075	0.051	3.380	2.495	0.026	0.053	2.334	0.041	2.997	2.368	0.019	4.304	4.191	0.128	3.305
P_{95}	*sel16786*	1.306	0.091	3.817	3.703	0.033	0.326	2.519	0.048	3.137	2.574	0.024	4.659	4.495	0.147	3.435
P_{50}	*sel16795*	1.693	0.058	3.110	2.482	0.057	1.388	2.440	0.025	4.069	2.789	0.031	0.223	4.039	0.134	3.810
P_5	*sel16795*	1.547	0.049	2.615	2.274	0.031	1.079	2.288	0.023	3.928	1.161	0.027	6.162	3.908	0.124	3.692
P_{95}	*sel16795*	1.794	0.072	4.058	2.714	0.087	2.234	2.556	0.027	4.494	4.526	0.050	1.054	4.287	0.144	3.859
P_{50}	*sel17152*	1.098	0.094	3.641	2.407	0.043	1.248	2.492	0.066	3.540	2.658	0.047	0.110	5.630	0.208	1.183
P_5	*sel17152*	1.047	0.083	3.369	2.369	0.019	1.062	2.450	0.060	3.414	1.941	0.010	5.695	5.527	0.156	0.518
P_{95}	*sel17152*	1.157	0.111	3.761	2.465	0.049	1.600	2.547	0.071	3.740	4.177	0.050	1.858	0.283	0.254	1.952
P_{50}	*sel17453*	1.487	0.068	3.285	2.436	0.048	1.192	2.437	0.035	3.613	2.175	0.036	5.940	4.402	0.157	3.438
P_5	*sel17453*	1.340	0.054	2.631	2.255	0.040	0.782	2.245	0.030	3.481	0.204	0.030	5.579	4.184	0.131	3.196
P_{95}	*sel17453*	1.676	0.099	3.989	2.647	0.051	1.401	2.604	0.038	3.702	2.948	0.044	0.036	4.712	0.204	3.687
P_{50}	*sel213*	0.951	0.119	3.232	2.313	0.057	1.543	2.483	0.090	3.693	2.727	0.033	0.798	5.388	0.332	2.149
P_5	*sel213*	0.756	0.075	2.643	2.070	0.035	5.774	2.340	0.055	3.477	2.226	0.010	4.729	5.136	0.260	1.603
P_{95}	*sel213*	1.141	0.156	5.335	2.611	0.119	2.179	2.620	0.198	3.961	3.440	0.067	2.225	5.577	0.388	2.722
P_{50}	*sel221*	0.913	0.248	3.030	2.371	0.040	1.830	2.473	0.056	3.364	2.495	0.022	5.767	4.837	0.233	2.496
P_5	*sel221*	0.435	0.077	1.770	2.038	0.028	1.214	2.153	0.049	3.292	2.181	0.009	4.557	4.308	0.179	2.085
P_{95}	*sel221*	1.466	0.390	4.243	2.625	0.055	2.537	2.754	0.062	3.426	3.240	0.027	0.143	5.268	0.295	3.128
P_{50}	*sel223*	1.024	0.106	2.944	2.319	0.079	1.535	2.453	0.069	3.618	2.950	0.044	0.495	5.262	0.190	1.446
P_5	*sel223*	0.904	0.069	2.580	2.245	0.044	1.205	2.390	0.051	3.522	2.644	0.019	6.084	4.998	0.136	0.915
P_{95}	*sel223*	1.119	0.143	3.583	2.456	0.106	1.820	2.523	0.081	3.666	3.275	0.050	0.810	5.547	0.226	1.909
P_{50}	*sel230*	1.158	0.097	4.025	2.378	0.048	2.135	2.425	0.038	3.876	2.922	0.031	0.345	4.226	0.264	3.280

	Patient	P Wave			Q Wave			R Wave			S Wave			T Wave		
		t^{FI}	ω	β	t^{FI}	ω	β	t^{FI}	ω	β	t^{FI}	ω	β	t^{FI}	ω	β
P_5	*sel230*	0.966	0.060	2.561	2.298	0.029	1.146	2.369	0.027	3.562	2.369	0.018	5.371	4.010	0.114	2.627
P_{95}	*sel230*	2.021	0.153	5.666	2.498	0.202	2.339	2.509	0.057	4.215	3.477	0.035	0.795	4.606	0.504	4.318
P_{50}	*sel231*	1.420	0.111	3.408	2.370	0.022	1.354	2.415	0.026	3.807	3.217	0.047	0.117	4.836	0.260	3.130
P_5	*sel231*	1.251	0.067	3.194	2.276	0.018	0.931	2.319	0.021	3.214	2.320	0.009	4.377	4.492	0.229	2.915
P_{95}	*sel231*	1.510	0.143	3.637	2.439	0.027	1.789	2.470	0.029	4.029	4.689	0.068	0.426	5.208	0.351	3.282
P_{50}	*sel232*	0.810	0.223	4.066	2.446	0.035	2.070	2.500	0.035	4.349	3.410	0.070	1.862	4.993	0.223	3.516
P_5	*sel232*	0.628	0.033	2.194	2.398	0.024	0.501	2.453	0.029	4.068	2.613	0.030	0.540	4.874	0.181	3.328
P_{95}	*sel232*	1.117	0.393	5.785	3.209	0.079	2.736	2.549	0.040	4.536	3.833	0.201	3.461	5.075	0.252	3.634
P_{50}	*sel233*	1.018	0.149	3.467	2.335	0.068	1.090	2.412	0.090	3.438	2.505	0.029	5.618	5.701	0.186	0.834
P_5	*sel233*	0.920	0.131	3.120	2.204	0.041	0.843	2.336	0.076	3.312	1.378	0.014	4.338	5.467	0.168	0.198
P_{95}	*sel233*	1.111	0.179	4.399	2.463	0.082	1.441	2.486	0.099	3.605	3.516	0.043	0.412	0.357	0.219	1.073
P_{50}	*sel30*	1.203	0.140	2.944	2.340	0.018	1.767	2.411	0.052	2.988	2.551	0.045	4.010	4.482	0.166	2.898
P_5	*sel30*	1.128	0.095	2.031	2.310	0.012	0.931	2.366	0.050	2.843	2.512	0.039	3.572	4.408	0.154	2.677
P_{95}	*sel30*	1.274	0.286	3.494	2.461	0.037	2.216	2.451	0.055	3.131	2.596	0.050	4.419	4.554	0.195	3.198
P_{50}	*sel301*	1.451	0.121	4.191	2.481	0.047	1.038	2.440	0.037	3.591	2.465	0.025	5.688	4.653	0.169	1.214
P_5	*sel301*	1.376	0.086	3.992	2.391	0.037	0.824	2.389	0.034	3.441	1.990	0.014	5.076	4.567	0.146	1.077
P_{95}	*sel301*	1.571	0.168	4.427	2.568	0.055	1.798	2.527	0.040	3.762	2.509	0.031	6.173	4.800	0.192	1.414
P_{50}	*sel302*	1.187	0.139	4.167	2.362	0.049	1.319	2.397	0.031	3.909	2.402	0.023	5.476	4.348	0.177	3.269
P_5	*sel302*	1.023	0.106	3.812	2.289	0.038	0.972	2.345	0.026	3.731	1.674	0.015	4.987	4.278	0.168	3.136
P_{95}	*sel302*	1.295	0.181	4.527	2.450	0.060	1.880	2.459	0.034	4.487	2.496	0.032	6.148	4.478	0.198	3.374
P_{50}	*sel306*	1.284	0.089	4.571	3.039	0.083	0.342	2.401	0.062	3.302	2.515	0.020	5.094	5.525	0.129	0.376
P_5	*sel306*	1.136	0.076	3.518	2.717	0.069	0.216	2.331	0.057	3.240	2.446	0.014	4.747	5.238	0.122	0.301
P_{95}	*sel306*	1.395	0.128	4.748	3.600	0.112	0.502	2.454	0.068	3.340	2.572	0.022	5.315	5.874	0.139	0.472
P_{50}	*sel307*	1.606	0.072	3.535	2.531	0.025	0.848	2.460	0.023	3.084	2.462	0.022	4.999	4.273	0.149	3.240
P_5	*sel307*	1.450	0.062	2.685	2.382	0.020	0.081	2.232	0.021	2.964	2.268	0.010	4.119	4.117	0.140	3.067
P_{95}	*sel307*	1.766	0.115	4.284	3.204	0.034	1.202	2.586	0.028	3.176	2.588	0.031	5.567	4.643	0.171	3.425
P_{50}	*sel308*	1.202	0.149	3.394	NA	NA	NA	2.148	0.049	2.934	6.021	0.135	6.232	4.330	0.119	3.140
P_5	*sel308*	1.063	0.090	2.723	NA	NA	NA	2.029	0.043	2.813	5.055	0.120	5.968	4.184	0.109	2.908
P_{95}	*sel308*	1.332	0.276	4.467	NA	NA	NA	2.226	0.054	3.115	1.247	0.156	0.089	4.418	0.129	3.329
P_{50}	*sel31*	1.019	0.156	3.907	2.370	0.060	2.234	2.460	0.074	3.937	2.952	0.049	1.500	5.943	0.249	3.017
P_5	*sel31*	0.807	0.113	3.529	2.302	0.039	1.174	2.430	0.061	3.657	2.576	0.022	4.871	5.305	0.088	0.155
P_{95}	*sel31*	1.093	0.253	4.675	2.469	0.103	3.409	2.488	0.082	4.098	3.037	0.241	4.045	1.578	0.406	5.180
P_{50}	*sel310*	0.240	0.187	2.623	2.241	0.040	1.546	2.385	0.054	3.257	2.366	0.048	5.376	4.647	0.312	2.810
P_5	*sel310*	0.169	0.138	2.207	2.212	0.025	0.606	2.354	0.041	3.144	1.895	0.022	4.624	4.581	0.288	2.615
P_{95}	*sel310*	0.340	0.238	2.981	2.413	0.050	2.116	2.433	0.063	3.428	2.520	0.050	6.032	4.699	0.332	3.074
P_{50}	*sel32*	1.241	0.093	3.958	2.475	0.047	0.635	2.364	0.067	3.013	2.631	0.044	5.689	0.850	0.179	0.166
P_5	*sel32*	1.178	0.069	3.330	2.300	0.028	0.129	2.317	0.060	2.753	1.702	0.029	4.934	5.989	0.129	6.129
P_{95}	*sel32*	1.329	0.127	4.331	3.515	0.084	1.506	2.416	0.075	3.215	3.528	0.117	1.745	2.475	0.251	0.609
P_{50}	*sel33*	1.996	0.055	3.338	2.413	0.024	0.665	2.451	0.027	2.973	2.470	0.024	5.189	4.467	0.265	3.717
P_5	*sel33*	1.830	0.032	2.181	1.411	0.010	5.218	2.305	0.020	2.666	2.127	0.012	4.442	4.194	0.194	3.232
P_{95}	*sel33*	2.229	0.080	5.023	2.643	0.105	1.719	2.596	0.030	3.370	3.504	0.033	0.244	4.687	0.391	4.567
P_{50}	*sel34*	1.507	0.141	2.384	2.430	0.024	0.385	2.441	0.041	2.979	2.499	0.023	4.436	4.305	0.185	2.977
P_5	*sel34*	0.637	0.068	1.621	1.422	0.010	5.672	2.328	0.033	2.674	2.386	0.018	4.172	4.026	0.114	2.537
P_{95}	*sel34*	1.763	0.361	4.007	2.765	0.034	1.436	2.543	0.047	3.461	2.812	0.039	0.103	4.510	0.264	3.684
P_{50}	*sel35*	0.983	0.097	2.324	2.362	0.041	0.750	2.468	0.091	3.097	2.893	0.053	5.256	5.070	0.072	1.630
P_5	*sel35*	0.030	0.044	0.205	0.072	0.022	5.920	2.043	0.064	2.648	2.085	0.025	3.746	3.627	0.044	5.651
P_{95}	*sel35*	1.967	0.221	4.654	2.814	0.077	2.134	2.805	0.152	3.443	4.064	0.232	1.486	6.148	0.151	4.321
P_{50}	*sel36*	1.051	0.184	3.534	2.333	0.048	1.458	2.465	0.056	4.227	3.127	0.063	0.883	5.453	0.344	3.899
P_5	*sel36*	6.033	0.098	3.024	2.266	0.029	0.768	2.422	0.039	3.767	2.398	0.031	5.176	5.311	0.179	3.499
P_{95}	*sel36*	1.595	0.384	6.235	2.461	0.060	2.737	2.508	0.064	4.357	3.602	0.128	1.750	5.577	0.557	4.432
P_{50}	*sel37*	0.982	0.123	5.885	2.374	0.038	1.219	2.477	0.037	3.627	2.545	0.028	5.885	4.911	0.135	1.789
P_5	*sel37*	4.769	0.039	3.301	1.563	0.016	6.070	2.434	0.024	2.900	1.413	0.012	4.281	3.996	0.071	6.222
P_{95}	*sel37*	2.367	0.348	2.385	2.865	0.069	1.802	2.594	0.069	4.518	4.070	0.171	1.594	5.647	0.341	4.008
P_{50}	*sel38*	6.176	0.191	5.077	1.868	0.070	1.788	2.026	0.096	3.241	5.332	0.242	0.107	4.944	0.309	3.011
P_5	*sel38*	4.931	0.090	3.286	1.746	0.061	1.234	1.931	0.078	3.045	4.709	0.228	6.276	4.795	0.191	2.230
P_{95}	*sel38*	0.159	0.327	5.870	2.036	0.122	2.190	2.085	0.101	3.947	5.858	0.257	0.339	5.066	0.379	3.633
P_{50}	*sel39*	1.559	0.129	3.220	1.995	0.045	1.658	2.400	0.042	2.640	2.548	0.048	5.311	4.931	0.197	2.014
P_5	*sel39*	1.097	0.086	0.940	1.227	0.017	4.957	2.017	0.028	2.327	2.144	0.035	4.570	4.414	0.133	0.931
P_{95}	*sel39*	1.851	0.264	4.234	2.534	0.101	4.521	2.624	0.062	4.181	2.863	0.065	5.844	0.070	0.307	4.101
P_{50}	*sel40*	2.640	0.305	0.503	1.931	0.040	2.924	2.166	0.047	4.325	3.812	0.129	0.300	4.066	0.262	3.624
P_5	*sel40*	0.306	0.025	4.718	1.814	0.023	1.840	2.052	0.036	3.354	3.448	0.114	0.124	4.019	0.149	3.140
P_{95}	*sel40*	4.867	0.393	2.822	2.011	0.100	3.612	2.272	0.096	4.912	4.553	0.142	0.565	4.262	0.371	4.218
P_{50}	*sel41*	0.114	0.203	4.687	2.058	0.087	0.685	2.014	0.052	3.131	4.359	0.116	0.143	3.260	0.287	6.159
P_5	*sel41*	5.867	0.138	3.611	1.952	0.031	4.678	1.985	0.029	2.589	3.426	0.097	6.139	1.368	0.155	4.158
P_{95}	*sel41*	1.483	0.347	6.039	3.060	0.267	2.009	2.063	0.077	4.139	0.257	0.128	0.328	5.416	0.534	2.207
P_{50}	*sel42*	1.871	0.056	0.671	1.943	0.030	5.867	2.371	0.164	3.333	3.387	0.237	2.135	5.691	0.196	0.541
P_5	*sel42*	5.963	0.040	5.313	1.768	0.019	4.771	2.276	0.133	2.813	2.339	0.063	5.731	3.894	0.166	6.023
P_{95}	*sel42*	3.410	0.087	3.203	2.383	0.064	2.075	2.427	0.235	3.542	3.470	0.299	2.638	1.162	0.244	0.851
P_{50}	*sel43*	1.482	0.083	1.559	2.297	0.069	1.441	2.417	0.099	3.402	2.742	0.048	2.254	5.743	0.315	1.082
P_5	*sel43*	0.003	0.039	5.886	2.189	0.041	0.818	2.383	0.084	3.194	2.610	0.019	1.656	5.330	0.139	0.269
P_{95}	*sel43*	2.423	0.185	4.123	2.445	0.105	2.049	2.453	0.119	3.657	2.846	0.082	3.131	0.145	0.477	2.910
P_{50}	*sel44*	0.692	0.113	2.470	2.445	0.049	0.898	2.418	0.075	3.359	2.623	0.035	4.550	5.372	0.385	2.081
P_5	*sel44*	0.460	0.057	0.956	1.588	0.029	5.197	1.896	0.061	3.029	2.369	0.013	2.348	5.027	0.222	0.561
P_{95}	*sel44*	1.426	0.189	3.779	2.624	0.100	1.503	2.660	0.088	3.733	2.896	0.170	5.174	0.055	0.511	3.362
P_{50}	*sel45*	0.735	0.117	2.500	2.417	0.076	1.197	2.413	0.043	4.188	3.107	0.030	0.240	5.363	0.407	3.794
P_5	*sel45*	0.641	0.061	1.332	2.301	0.045	0.781	2.354	0.031	4.062	2.748	0.021	0.010	4.598	0.194	1.412
P_{95}	*sel45*	0.904	0.160	3.492	2.792	0.113	1.980	2.460	0.049	4.435	4.640	0.037	1.344	5.492	0.630	5.915
P_{50}	*sel46*	0.713	0.102	4.155	2.340	0.030	2.085	2.424	0.045	4.195	2.783	0.035	0.834	4.970	0.149	0.970
P_5	*sel46*	4.969	0.041	1.819	2.186	0.023	1.692	2.314	0.033	2.630	2.268	0.023	5.901	2.628	0.108	5.988
P_{95}	*sel46*	2.429	0.270	0.082	2.487	0.041	2.657	2.568	0.059	4.487	2.976	0.066	1.545	0.305	0.254	2.183

		P Wave			Q Wave			R Wave			S Wave			T Wave		
	Patient	t^{FI}	ω	β	t^{FI}	ω	β	t^{FI}	ω	β	t^{FI}	ω	β	t^{FI}	ω	β
P_{50}	*sel47*	1.311	0.121	2.254	2.354	0.037	1.976	2.411	0.038	3.829	2.841	0.040	0.708	5.623	0.356	1.113
P_5	*sel47*	1.075	0.062	0.943	1.957	0.021	5.698	2.360	0.027	3.173	2.028	0.023	5.530	5.327	0.276	0.067
P_{95}	*sel47*	1.434	0.255	4.274	3.268	0.195	3.039	2.491	0.052	4.195	3.805	0.109	1.910	0.789	0.450	2.559
P_{50}	*sel48*	0.935	0.188	2.900	2.330	0.055	0.886	2.416	0.093	3.489	2.773	0.048	3.730	5.393	0.298	1.584
P_5	*sel48*	0.902	0.108	2.396	2.234	0.047	0.717	2.389	0.082	3.185	2.664	0.026	2.708	5.129	0.181	1.251
P_{95}	*sel48*	1.023	0.243	3.670	2.436	0.073	1.406	2.445	0.109	3.577	2.920	0.079	5.313	5.554	0.355	2.143
P_{50}	*sel49*	1.611	0.048	3.512	2.360	0.039	1.975	2.478	0.052	3.469	2.502	0.019	5.750	4.587	0.168	1.429
P_5	*sel49*	1.548	0.035	2.166	2.341	0.032	1.631	2.462	0.045	3.369	0.964	0.013	5.197	4.491	0.133	1.055
P_{95}	*sel49*	1.653	0.074	4.684	2.380	0.044	2.272	2.499	0.054	3.567	3.269	0.035	0.267	4.790	0.239	1.773
P_{50}	*sel50*	1.591	0.160	2.399	2.460	0.032	1.619	2.497	0.047	3.899	3.000	0.030	0.615	4.750	0.178	1.074
P_5	*sel50*	0.498	0.051	0.778	1.869	0.025	1.202	1.991	0.024	3.555	2.129	0.020	5.837	4.046	0.144	0.634
P_{95}	*sel50*	2.481	0.391	4.781	3.153	0.071	2.429	3.162	0.071	4.284	4.864	0.088	1.641	0.079	0.396	1.679
P_{50}	*sel51*	1.456	0.068	3.769	2.438	0.036	1.709	2.485	0.037	3.447	2.438	0.023	5.675	4.437	0.118	1.786
P_5	*sel51*	1.357	0.056	3.331	2.396	0.030	0.549	2.436	0.033	3.241	2.343	0.020	5.074	4.326	0.092	1.448
P_{95}	*sel51*	1.517	0.113	4.054	2.853	0.069	2.044	2.545	0.044	3.560	2.498	0.025	5.885	5.210	0.550	3.465
P_{50}	*sel52*	0.419	0.298	3.878	2.322	0.040	1.898	2.424	0.058	3.580	2.510	0.034	0.623	5.192	0.113	1.199
P_5	*sel52*	0.287	0.137	2.606	2.283	0.031	1.130	2.385	0.047	3.357	2.140	0.009	5.048	4.104	0.073	0.197
P_{95}	*sel52*	0.607	0.394	4.665	2.552	0.076	2.619	2.465	0.066	3.729	3.853	0.247	2.041	6.248	0.166	2.508
P_{50}	*sel803*	1.229	0.084	3.813	2.396	0.030	1.553	2.452	0.034	3.172	2.405	0.025	5.826	4.337	0.161	3.682
P_5	*sel803*	1.203	0.069	3.602	2.358	0.025	1.330	2.411	0.029	3.078	1.658	0.022	5.505	4.286	0.155	3.549
P_{95}	*sel803*	1.310	0.100	3.965	2.438	0.034	1.891	2.483	0.038	3.304	2.476	0.029	6.193	4.411	0.180	3.788
P_{50}	*sel808*	1.454	0.090	3.584	2.614	0.034	0.300	2.442	0.055	3.395	2.567	0.020	5.056	4.967	0.127	0.626
P_5	*sel808*	1.368	0.072	1.972	2.409	0.029	0.150	2.423	0.050	3.338	2.509	0.015	4.465	4.839	0.112	0.421
P_{95}	*sel808*	1.513	0.144	3.859	3.052	0.042	0.914	2.476	0.060	3.562	2.638	0.025	5.749	5.261	0.165	1.215
P_{50}	*sel811*	1.644	0.106	4.011	2.413	0.017	1.188	2.453	0.032	3.085	2.509	0.018	4.861	4.000	0.129	3.485
P_5	*sel811*	1.556	0.073	3.393	2.380	0.013	0.862	2.371	0.030	2.817	2.446	0.015	4.632	3.907	0.124	3.295
P_{95}	*sel811*	1.735	0.192	4.242	2.545	0.020	1.714	2.536	0.034	3.220	2.604	0.022	5.066	4.111	0.142	3.619
P_{50}	*sel820*	1.066	0.230	3.144	2.392	0.041	0.823	2.406	0.072	3.514	2.540	0.029	4.154	5.151	0.342	1.677
P_5	*sel820*	1.031	0.170	2.872	2.336	0.037	0.655	2.377	0.065	3.336	2.505	0.008	3.532	5.037	0.300	1.545
P_{95}	*sel820*	1.111	0.302	3.368	2.450	0.043	1.039	2.462	0.078	3.662	2.581	0.043	4.715	5.248	0.398	1.865
P_{50}	*sel821*	0.741	0.110	3.591	2.116	0.042	1.794	2.326	0.042	3.382	3.464	0.061	0.246	4.857	0.224	3.407
P_5	*sel821*	0.139	0.054	2.229	1.522	0.027	1.172	1.618	0.030	3.098	2.608	0.043	6.032	3.980	0.191	3.111
P_{95}	*sel821*	1.208	0.176	4.394	2.377	0.176	2.122	2.609	0.054	3.797	5.532	0.073	0.491	5.583	0.304	3.753
P_{50}	*sel840*	1.198	0.084	3.613	2.367	0.029	1.784	2.389	0.031	3.705	2.342	0.031	6.121	4.569	0.212	3.485
P_5	*sel840*	0.147	0.027	2.224	2.188	0.014	1.319	2.267	0.020	2.251	0.769	0.023	5.333	4.394	0.184	3.305
P_{95}	*sel840*	1.491	0.386	4.715	2.474	0.046	2.328	2.495	0.037	4.429	4.242	0.039	0.383	4.661	0.262	3.705
P_{50}	*sel847*	1.354	0.062	3.038	2.410	0.035	1.265	2.447	0.035	2.979	2.496	0.033	5.155	4.049	0.161	3.221
P_5	*sel847*	1.250	0.043	2.139	2.342	0.025	0.873	2.388	0.028	2.769	2.437	0.014	4.547	3.972	0.145	3.091
P_{95}	*sel847*	1.446	0.104	3.943	2.463	0.049	1.685	2.478	0.041	3.431	2.550	0.040	5.315	4.158	0.172	3.300
P_{50}	*sel853*	1.428	0.091	2.912	2.334	0.071	0.951	2.347	0.068	4.249	2.958	0.056	0.799	5.261	0.163	0.833
P_5	*sel853*	1.311	0.056	1.687	2.223	0.046	0.806	2.294	0.050	4.082	2.471	0.030	0.179	4.984	0.110	0.352
P_{95}	*sel853*	2.355	0.163	4.379	2.467	0.087	1.214	2.400	0.078	4.430	3.093	0.069	1.468	5.891	0.182	1.208
P_{50}	*sel871*	1.309	0.071	3.611	2.229	0.013	0.436	2.280	0.036	3.031	1.062	0.043	6.204	4.279	0.165	3.529
P_5	*sel871*	1.212	0.047	2.962	1.769	0.006	5.520	2.226	0.027	2.629	4.891	0.031	5.910	3.997	0.130	3.165
P_{95}	*sel871*	1.436	0.109	4.544	2.709	0.038	1.668	2.342	0.048	3.923	2.564	0.048	0.099	4.442	0.198	3.878
P_{50}	*sel872*	1.116	0.090	3.860	2.402	0.030	1.447	2.449	0.036	3.162	2.394	0.025	5.780	4.294	0.161	3.659
P_5	*sel872*	1.063	0.078	3.690	2.333	0.024	1.195	2.389	0.031	3.072	2.230	0.021	5.482	4.217	0.156	3.476
P_{95}	*sel872*	1.159	0.117	4.031	2.461	0.036	1.868	2.513	0.039	3.320	2.473	0.028	6.108	4.387	0.169	3.763
P_{50}	*sel873*	1.258	0.116	4.596	2.362	0.029	1.346	2.457	0.045	3.126	2.545	0.030	5.047	4.111	0.178	2.672
P_5	*sel873*	5.764	0.050	2.745	2.287	0.016	0.703	2.394	0.037	2.876	2.454	0.023	4.421	4.028	0.147	1.500
P_{95}	*sel873*	1.422	0.366	5.615	2.442	0.041	2.182	2.500	0.057	3.325	2.618	0.033	5.297	4.250	0.242	3.140
P_{50}	*sel883*	1.314	0.070	5.858	2.568	0.047	0.628	2.445	0.055	3.676	2.580	0.013	5.700	5.529	0.169	0.696
P_5	*sel883*	6.231	0.045	4.779	2.411	0.033	0.300	2.390	0.042	3.456	1.994	0.008	4.084	5.253	0.148	0.415
P_{95}	*sel883*	1.628	0.144	2.487	2.813	0.070	0.902	2.488	0.060	3.831	3.051	0.020	0.666	6.043	0.204	1.059
P_{50}	*sel891*	0.118	0.118	3.781	2.205	0.034	1.894	2.306	0.050	2.646	2.307	0.075	5.469	4.866	0.203	3.249
P_5	*sel891*	5.129	0.057	2.794	1.489	0.011	1.158	1.569	0.029	2.273	0.840	0.030	4.749	3.864	0.162	2.958
P_{95}	*sel891*	1.085	0.340	6.240	2.649	0.125	2.465	2.694	0.082	4.202	3.810	0.108	0.283	5.719	0.258	3.783

Table S5: Individual performance measures for P wave detection in QT database. NA is assigned when P wave is not annotated. Patients are sorted alphabetically

	P Wave							
Patient	N^o beats	TP	FP	FN	Se(%)	PPV(%)	DER(%)	F1(%)
sel0104	30	30	0	0	100	100	0	100
sel0106	30	30	0	0	100	100	0	100
sel0107	34	34	0	0	100	100	0	100
sel0110	30	23	5	7	76.667	82.143	40	79.31
sel0111	30	30	0	0	100	100	0	100
sel0112	50	41	6	9	82	87.234	30	84.536
sel0114	30	26	5	4	86.667	83.871	30	85.246
sel0116	30	15	10	15	50	60	83.333	54.545
sel0121	30	30	0	0	100	100	0	100
sel0122	30	30	0	0	100	100	0	100
sel0124	50	50	0	0	100	100	0	100
sel0126	30	30	2	0	100	93.75	6.667	96.774
sel0129	30	21	6	9	70	77.778	50	73.684

P Wave

Patient	N^o beats	TP	FP	FN	Se(%)	PPV(%)	DER(%)	F1(%)
sel0133	30	30	3	0	100	90.909	10	95.238
sel0136	30	30	0	0	100	100	0	100
sel0166	36	36	0	0	100	100	0	100
sel0170	30	30	0	0	100	100	0	100
sel0203	30	30	0	0	100	100	0	100
sel0210	30	30	0	0	100	100	0	100
sel0211	30	30	0	0	100	100	0	100
sel0303	30	30	0	0	100	100	0	100
sel0405	30	30	2	0	100	93.75	6.667	96.774
sel0406	31	31	5	0	100	86.111	16.129	92.537
sel0409	30	30	0	0	100	100	0	100
sel0411	30	30	0	0	100	100	0	100
sel0509	30	30	13	0	100	69.767	43.333	82.192
sel0603	30	30	0	0	100	100	0	100
sel0604	30	30	0	0	100	100	0	100
sel0606	30	30	3	0	100	90.909	10	95.238
sel0607	30	30	0	0	100	100	0	100
sel0609	30	30	0	0	100	100	0	100
sel0612	30	30	0	0	100	100	0	100
sel0704	30	30	0	0	100	100	0	100
sel100	30	30	0	0	100	100	0	100
sel102	0	0	0	0	NA	NA	NA	NA
sel103	30	30	0	0	100	100	0	100
sel104	17	17	0	0	100	100	0	100
sel114	50	48	5	2	96	90.566	14	93.204
sel116	50	49	0	1	98	100	2	98.99
sel117	30	30	0	0	100	100	0	100
sel123	30	30	0	0	100	100	0	100
sel14046	31	27	2	4	87.097	93.103	19.355	90
sel14157	28	28	1	0	100	96.552	3.571	98.246
sel14172	50	49	5	1	98	90.741	12	94.231
sel15814	30	30	0	0	100	100	0	100
sel16265	30	30	0	0	100	100	0	100
sel16272	30	30	0	0	100	100	0	100
sel16273	30	30	1	0	100	96.774	3.333	98.361
sel16420	30	30	0	0	100	100	0	100
sel16483	30	30	0	0	100	100	0	100
sel16539	30	30	0	0	100	100	0	100
sel16773	30	29	0	1	96.667	100	3.333	98.305
sel16786	30	30	0	0	100	100	0	100
sel16795	30	30	0	0	100	100	0	100
sel17152	30	30	0	0	100	100	0	100
sel17453	30	30	0	0	100	100	0	100
sel213	71	71	5	0	100	93.421	7.042	96.599
sel221	0	0	0	0	NA	NA	NA	NA
sel223	31	31	1	0	100	96.875	3.226	98.413
sel230	50	50	1	0	100	98.039	2	99.01
sel231	50	50	0	0	100	100	0	100
sel232	0	0	0	0	NA	NA	NA	NA
sel233	30	30	0	0	100	100	0	100
sel30	30	30	0	0	100	100	0	100
sel301	30	30	0	0	100	100	0	100
sel302	30	30	0	0	100	100	0	100
sel306	36	36	0	0	100	100	0	100
sel307	30	30	0	0	100	100	0	100
sel308	50	50	0	0	100	100	0	100
sel31	30	30	2	0	100	93.75	6.667	96.774
sel310	0	0	0	0	NA	NA	NA	NA
sel32	30	30	0	0	100	100	0	100
sel33	30	29	2	1	96.667	93.548	10	95.082
sel34	30	29	4	1	96.667	87.879	16.667	92.063
sel35	0	0	0	0	NA	NA	NA	NA
sel36	1	1	1	0	100	50	100	66.667
sel37	0	0	0	0	NA	NA	NA	NA
sel38	30	30	0	0	100	100	0	100
sel39	30	30	1	0	100	96.774	3.333	98.361
sel40	30	30	13	0	100	69.767	43.333	82.192
sel41	30	30	10	0	100	75	33.333	85.714
sel42	30	30	21	0	100	58.824	70	74.074
sel43	30	30	16	0	100	65.217	53.333	78.947
sel44	22	22	0	0	100	100	0	100
sel45	30	30	3	0	100	90.909	10	95.238
sel46	29	29	8	0	100	78.378	27.586	87.879
sel47	30	30	1	0	100	96.774	3.333	98.361
sel48	30	30	0	0	100	100	0	100
sel49	30	30	0	0	100	100	0	100
sel50	0	0	0	0	NA	NA	NA	NA
sel51	30	30	0	0	100	100	0	100
sel52	30	30	1	0	100	96.774	3.333	98.361
sel803	30	30	0	0	100	100	0	100

				P Wave				
Patient	N^o beats	TP	FP	FN	Se(%)	PPV(%)	DER(%)	F1(%)
sel808	30	30	0	0	100	100	0	100
sel811	30	30	0	0	100	100	0	100
sel820	30	30	0	0	100	100	0	100
sel821	26	26	0	0	100	100	0	100
sel840	47	26	8	21	55.319	76.471	61.702	64.198
sel847	33	33	0	0	100	100	0	100
sel853	30	26	1	4	86.667	96.296	16.667	91.228
sel871	70	70	1	0	100	98.592	1.429	99.291
sel872	30	30	0	0	100	100	0	100
sel873	33	20	8	13	60.606	71.429	63.636	65.574
sel883	30	29	19	1	96.667	60.417	66.667	74.359
sel891	58	43	11	15	74.138	79.63	44.828	76.786

Table S6: Individual performance measures for *T* wave detection in QT database. NA is assigned when *T* wave is not annotated. Patients are sorted alphabetically

					T Wave			
Patient	N^o beats	TP	FP	FN	Se(%)	PPV(%)	DER(%)	F1(%)
sel0104	30	30	1	0	100	96.774	3.333	98.361
sel0106	30	30	0	0	100	100	0	100
sel0107	34	34	0	0	100	100	0	100
sel0110	30	30	0	0	100	100	0	100
sel0111	30	30	0	0	100	100	0	100
sel0112	50	50	0	0	100	100	0	100
sel0114	30	30	0	0	100	100	0	100
sel0116	30	30	0	0	100	100	0	100
sel0121	30	30	0	0	100	100	0	100
sel0122	30	30	0	0	100	100	0	100
sel0124	50	50	0	0	100	100	0	100
sel0126	30	30	0	0	100	100	0	100
sel0129	30	30	0	0	100	100	0	100
sel0133	30	30	0	0	100	100	0	100
sel0136	30	30	0	0	100	100	0	100
sel0166	36	36	0	0	100	100	0	100
sel0170	30	30	0	0	100	100	0	100
sel0203	30	30	0	0	100	100	0	100
sel0210	30	30	0	0	100	100	0	100
sel0211	30	30	0	0	100	100	0	100
sel0303	30	30	0	0	100	100	0	100
sel0405	30	30	0	0	100	100	0	100
sel0406	31	31	0	0	100	100	0	100
sel0409	30	30	27	0	100	52.632	90	68.966
sel0411	30	30	0	0	100	100	0	100
sel0509	30	30	0	0	100	100	0	100
sel0603	30	30	0	0	100	100	0	100
sel0604	30	30	0	0	100	100	0	100
sel0606	30	30	0	0	100	100	0	100
sel0607	30	30	3	0	100	90.909	10	95.238
sel0609	30	30	0	0	100	100	0	100
sel0612	30	30	0	0	100	100	0	100
sel0704	30	30	0	0	100	100	0	100
sel100	30	30	22	0	100	57.692	73.333	73.171
sel102	85	85	12	0	100	87.629	14.118	93.407
sel103	30	30	0	0	100	100	0	100
sel104	77	77	6	0	100	92.771	7.792	96.25
sel114	50	50	5	0	100	90.909	10	95.238
sel116	50	50	0	0	100	100	0	100
sel117	30	30	0	0	100	100	0	100
sel123	30	30	0	0	100	100	0	100
sel14046	31	31	0	0	100	100	0	100
sel14157	30	30	0	0	100	100	0	100
sel14172	50	50	45	0	100	52.632	90	68.966
sel15814	30	30	0	0	100	100	0	100
sel16265	30	30	0	0	100	100	0	100
sel16272	30	30	0	0	100	100	0	100
sel16273	30	30	0	0	100	100	0	100
sel16420	30	30	0	0	100	100	0	100
sel16483	30	30	0	0	100	100	0	100
sel16539	30	30	0	0	100	100	0	100
sel16773	30	30	0	0	100	100	0	100
sel16786	30	30	0	0	100	100	0	100
sel16795	30	30	0	0	100	100	0	100
sel17152	30	30	6	0	100	83.333	20	90.909
sel17453	30	30	0	0	100	100	0	100
sel213	71	71	39	0	100	64.545	54.93	78.453
sel221	30	30	0	0	100	100	0	100

T Wave								
Patient	N^o beats	TP	FP	FN	Se(%)	PPV(%)	DER(%)	F1(%)
sel223	31	31	12	0	100	72.093	38.71	83.784
sel230	50	50	0	0	100	100	0	100
sel231	50	50	0	0	100	100	0	100
sel232	30	30	0	0	100	100	0	100
sel233	30	30	0	0	100	100	0	100
sel30	30	30	0	0	100	100	0	100
sel301	30	30	28	0	100	51.724	93.333	68.182
sel302	30	30	0	0	100	100	0	100
sel306	36	36	0	0	100	100	0	100
sel307	30	30	0	0	100	100	0	100
sel308	50	50	0	0	100	100	0	100
sel31	30	30	20	0	100	60	66.667	75
sel310	30	30	0	0	100	100	0	100
sel32	30	30	1	0	100	96.774	3.333	98.361
sel33	30	30	0	0	100	100	0	100
sel34	30	30	0	0	100	100	0	100
sel35	0	0	0	0	NA	NA	NA	NA
sel36	31	31	26	0	100	54.386	83.871	70.455
sel37	0	0	0	0	NA	NA	NA	NA
sel38	30	30	0	0	100	100	0	100
sel39	30	30	7	0	100	81.081	23.333	89.552
sel40	30	30	0	0	100	100	0	100
sel41	30	30	27	0	100	52.632	90	68.966
sel42	30	30	8	0	100	78.947	26.667	88.235
sel43	30	30	8	0	100	78.947	26.667	88.235
sel44	30	30	9	0	100	76.923	30	86.957
sel45	30	30	26	0	100	53.571	86.667	69.767
sel46	30	30	8	0	100	78.947	26.667	88.235
sel47	30	30	6	0	100	83.333	20	90.909
sel48	30	30	13	0	100	69.767	43.333	82.192
sel49	30	30	8	0	100	78.947	26.667	88.235
sel50	32	32	2	0	100	94.118	6.25	96.97
sel51	30	30	13	0	100	69.767	43.333	82.192
sel52	30	30	1	0	100	96.774	3.333	98.361
sel803	30	30	0	0	100	100	0	100
sel808	30	30	0	0	100	100	0	100
sel811	30	30	0	0	100	100	0	100
sel820	30	30	26	0	100	53.571	86.667	69.767
sel821	30	30	0	0	100	100	0	100
sel840	70	70	0	0	100	100	0	100
sel847	33	33	0	0	100	100	0	100
sel853	30	30	0	0	100	100	0	100
sel871	70	70	0	0	100	100	0	100
sel872	30	30	0	0	100	100	0	100
sel873	33	33	0	0	100	100	0	100
sel883	30	30	0	0	100	100	0	100
sel891	71	71	0	0	100	100	0	100

2.2 Patient identification analysis

Next, patient identification is deeply analysed in order to reinforce FMM_{ecg} discriminating ability. First, to accomplish error rate given for the whole Fisher LDA on the whole QT database (see Subsection 2.2.1 of the main text), the related confusion matrix is computed. In view of the limited space, only patient-specific diagonal values are gathered in Table S7. In addition to this, Fisher LDA has been separately conducted on each of the subgroups of the QT database defined according to the source of the data. Table S8 describes the the original database for the seven QT subgroups considered and the patient distribution in each of them, one may refer to (`https://physionet.org`) for details. LDA subgroup analyses were performed similar to those given for the whole data set in the main text. Fisher LDA is applied, using $A_J, \omega_J, \beta_J, J = P, Q, R, S, T$ as predictors and the one-leave-out rule to estimate the error rate. Subgroups error rates are shown in Table S9. It is observed that the error rate percentage is lower than 2% for 4 out of the 7 subgroups considered that gather more than 50% of the patients in QT

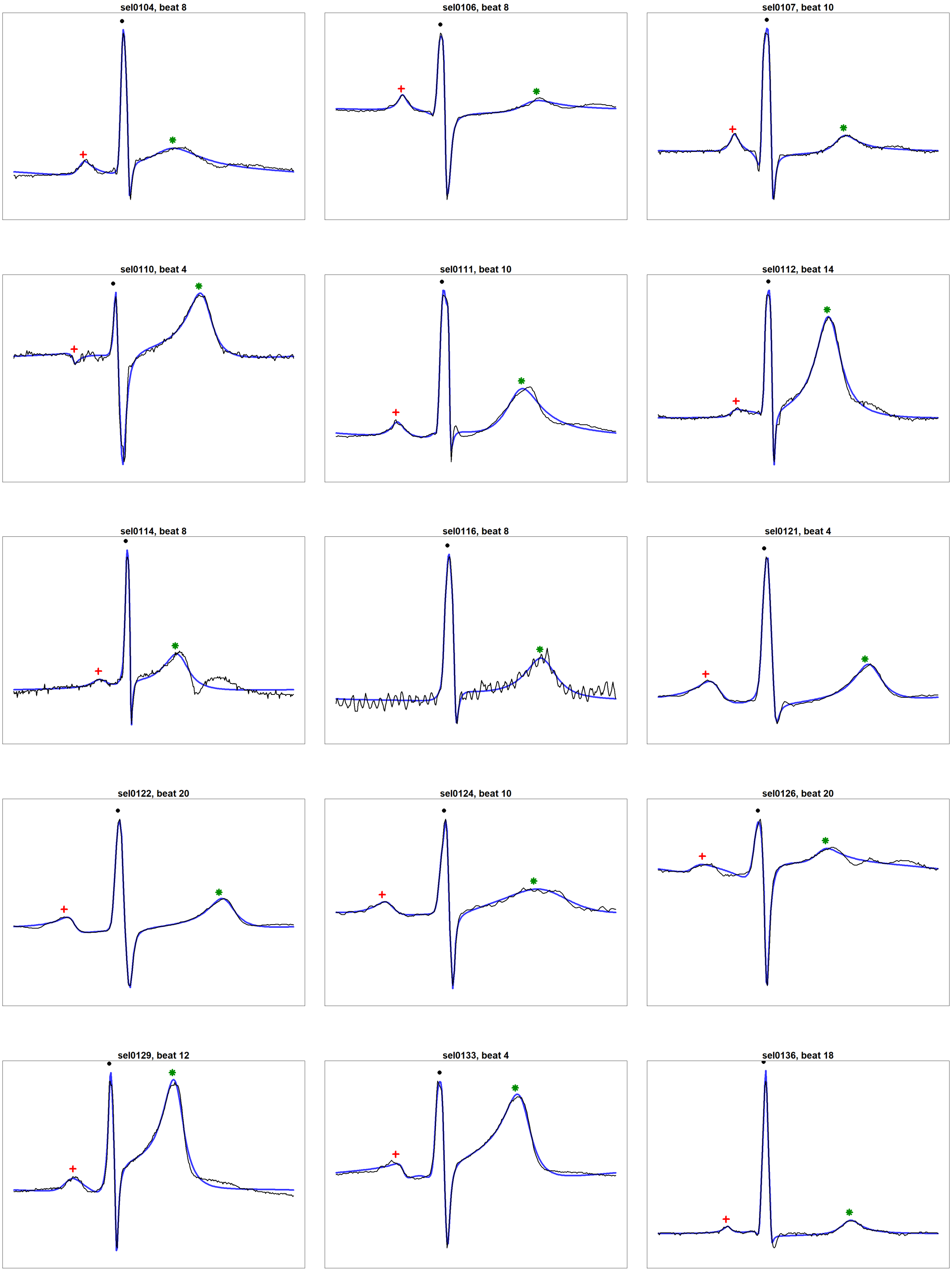

Figure S4: Representative beats (black line) for patients *sel0104*, *sel0106*, *sel0107*, *sel0110*, *sel0111*, *sel0112*, *sel0114*, *sel0116*, *sel0121*, *sel0122*, *sel0124*, *sel0126*, *sel0129*, *sel0133*, *sel0136*; FMM_{ecg} fits (blue line) and fiducial marks for R wave($\bullet$), T wave($\star$), P wave($+$). Patients are sorted alphabetically

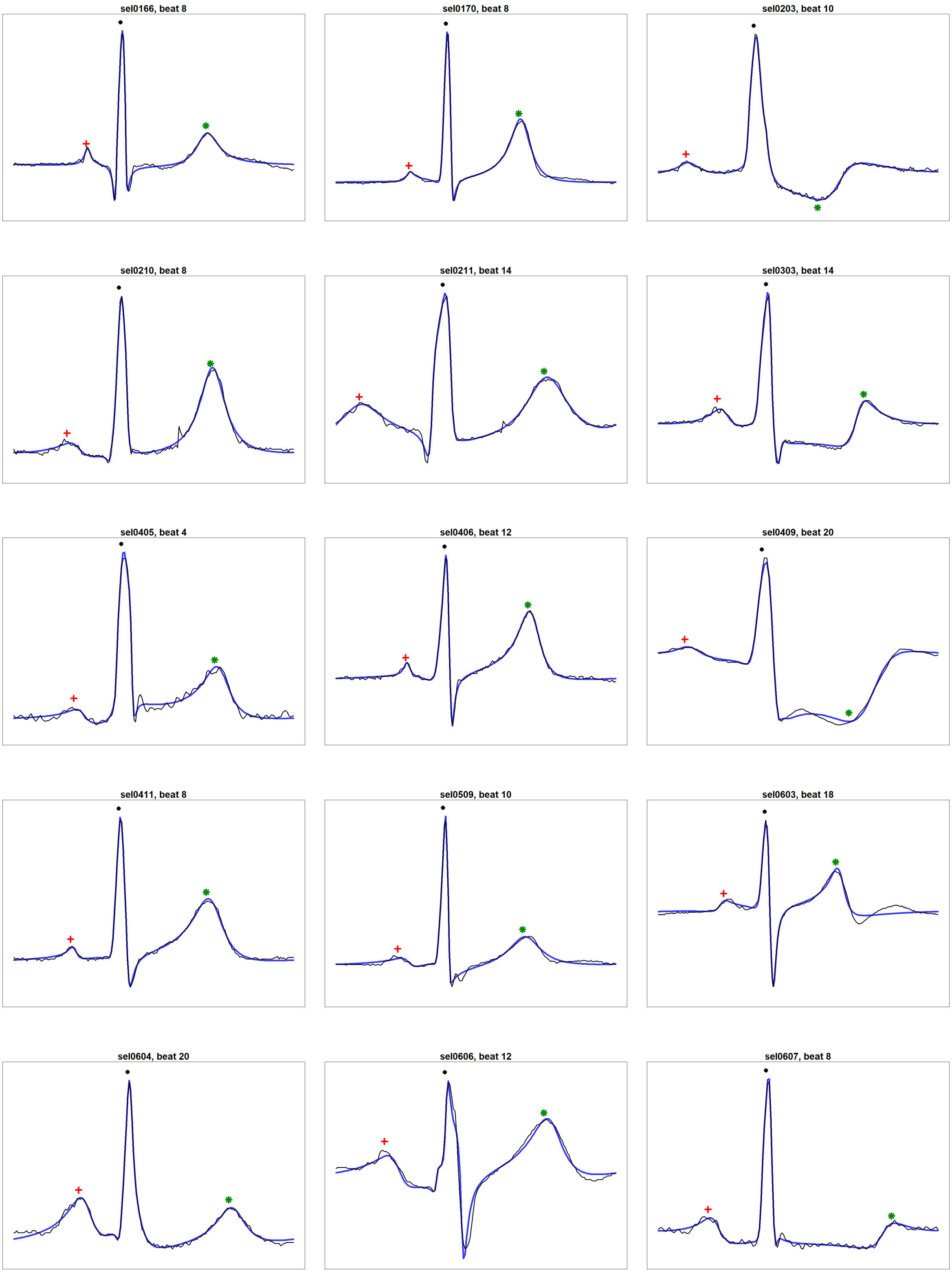

Figure S5: Representative beats (black line) for patients *sel0166*, *sel0170*, *sel0203*, *sel0210*, *sel0211*, *sel0303*, *sel0405*, *sel0406*, *sel0409*, *sel0411*, *sel0509*, *sel0603*, *sel0604*, *sel0606*, *sel0607*; FMM_{ecg} fits (blue line) and fiducial marks for R wave($\bullet$), T wave($\star$), P wave($+$). Patients are sorted alphabetically

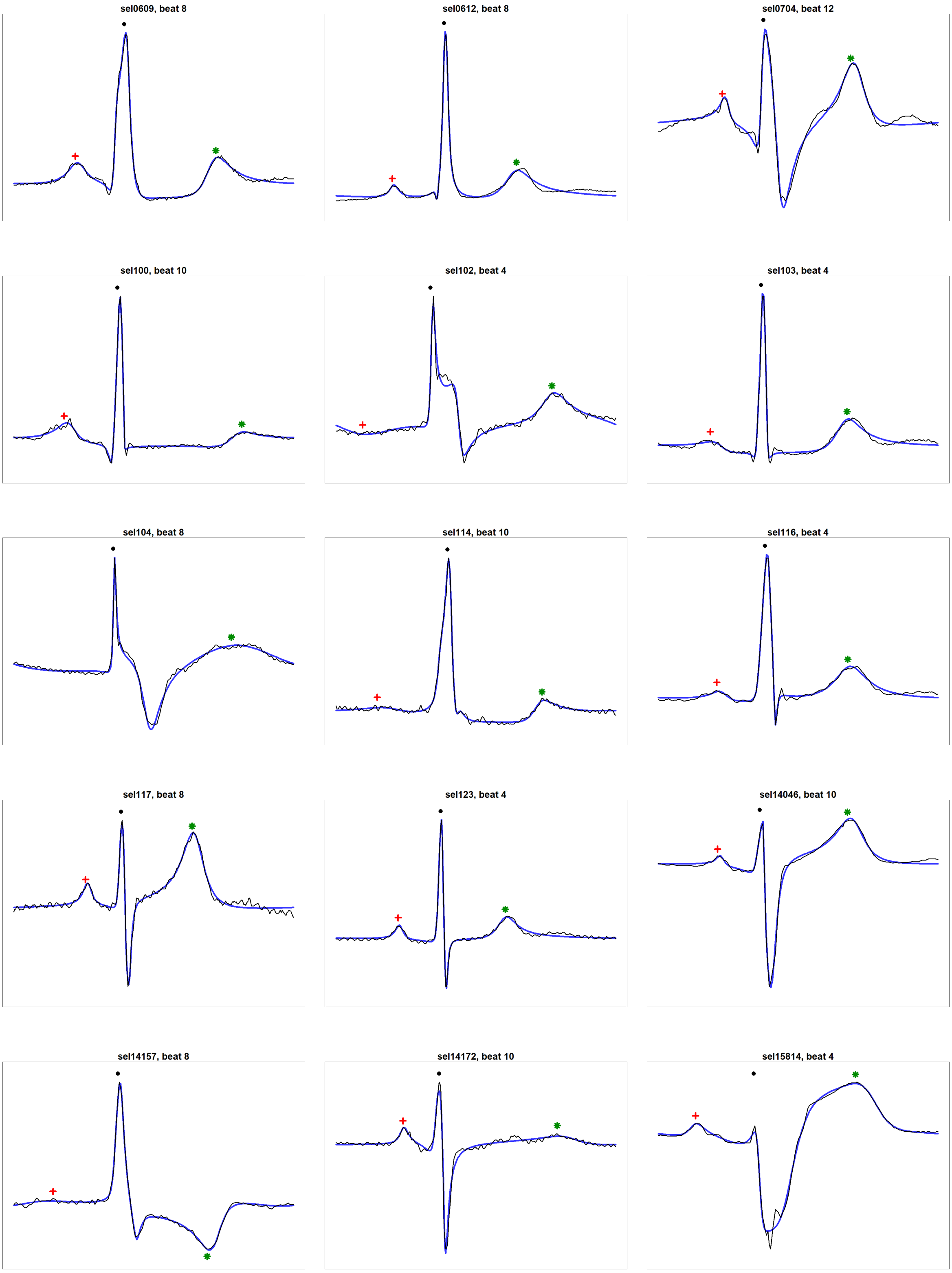

Figure S6: Representative beats (black line) for patients *sel0609*, *sel0612*, *sel0714*, *sel100*, *sel102*, *sel103*, *sel104*, *sel114*, *sel116*, *sel117*, *sel123*, *sel14046*, *sel14157*, *sel14172*, *sel15814*; FMM_{ecg} fits (blue line) and fiducial marks for R wave(●), T wave($\star$), P wave($+$). Patients are sorted alphabetically

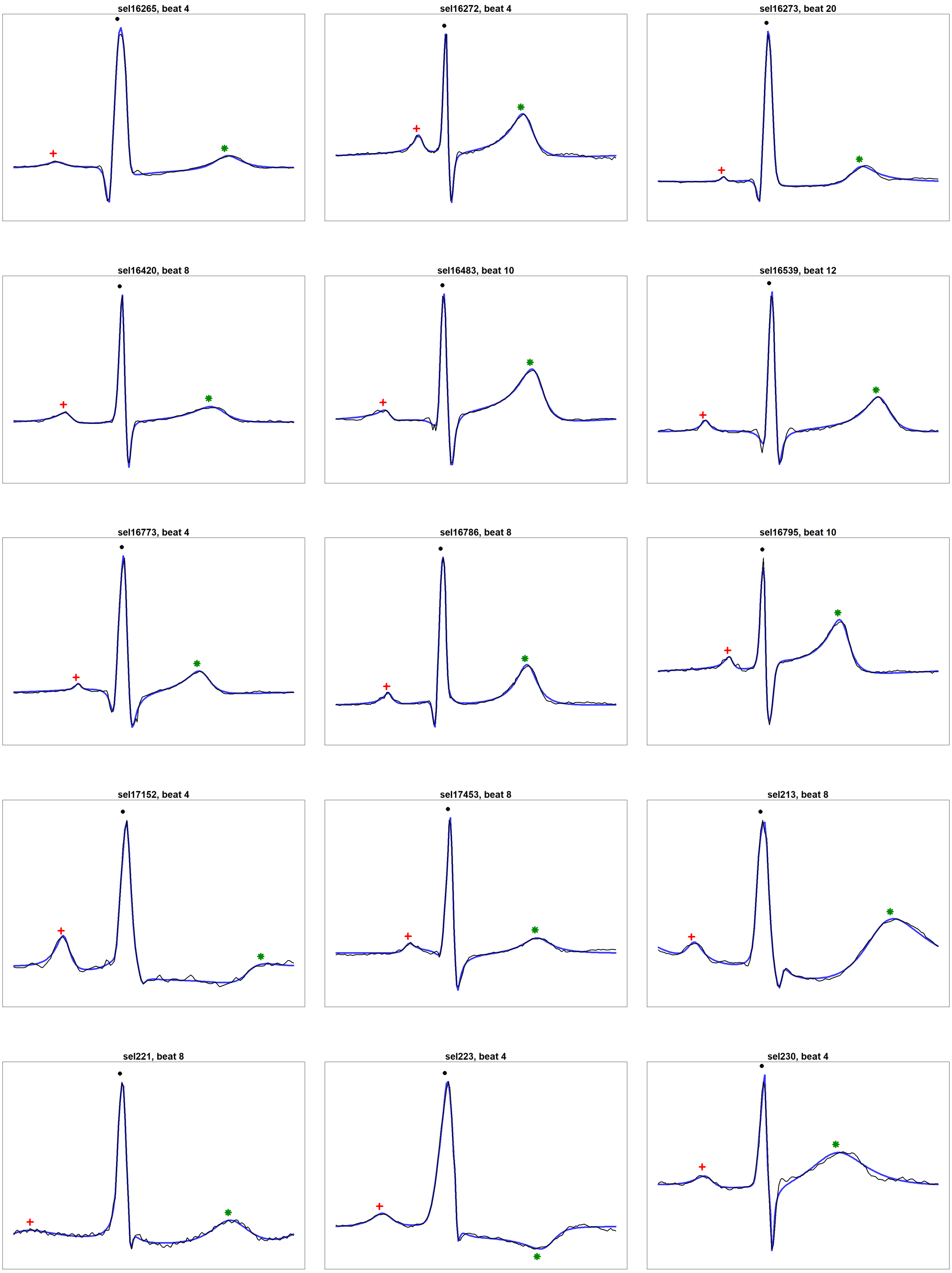

Figure S7: Representative beats (black line) for patients *sel16265*, *sel16272*, *sel16273*, *sel16420*, *sel16483*, *sel16539*, *sel16773*, *sel16786*, *sel16795*, *sel17152*, *sel17453*, *sel213*, *sel221*, *sel223*, *sel230*; FMM_{ecg} fits (blue line) and fiducial marks for R wave(●), T wave($\star$), P wave($+$). Patients are sorted alphabetically

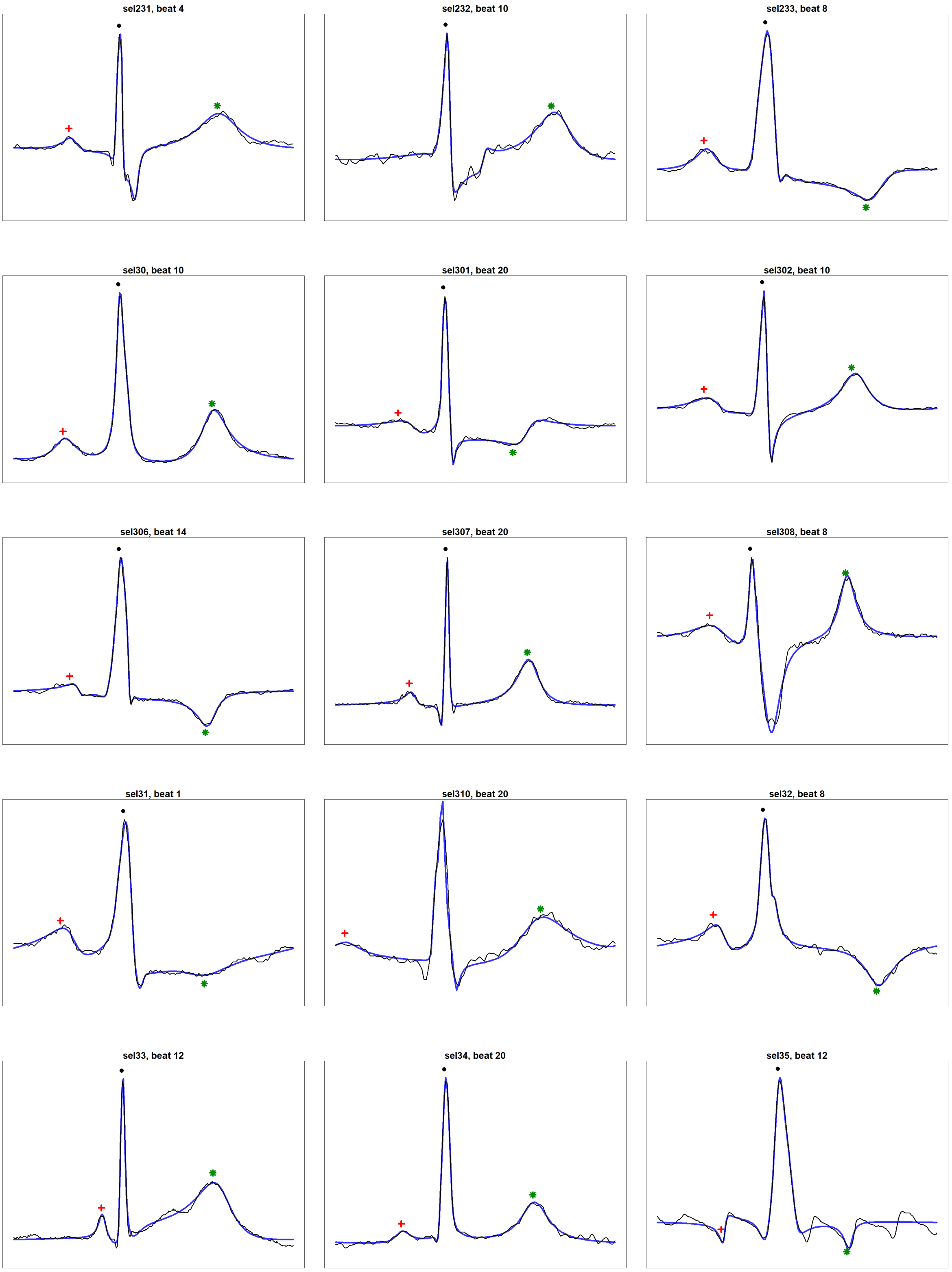

Figure S8: Representative beats (black line) for patients *sel231*, *sel232*, *sel233*, *sel30*, *sel301*, *sel302*, *sel306*, *sel307*, *sel308*, *sel31*, *sel310*, *sel32*, *sel33*, *sel34*, *sel35*; FMM_{ecg} fits (blue line) and fiducial marks for R wave($\bullet$), T wave($\star$), P wave($+$). Patients are sorted alphabetically

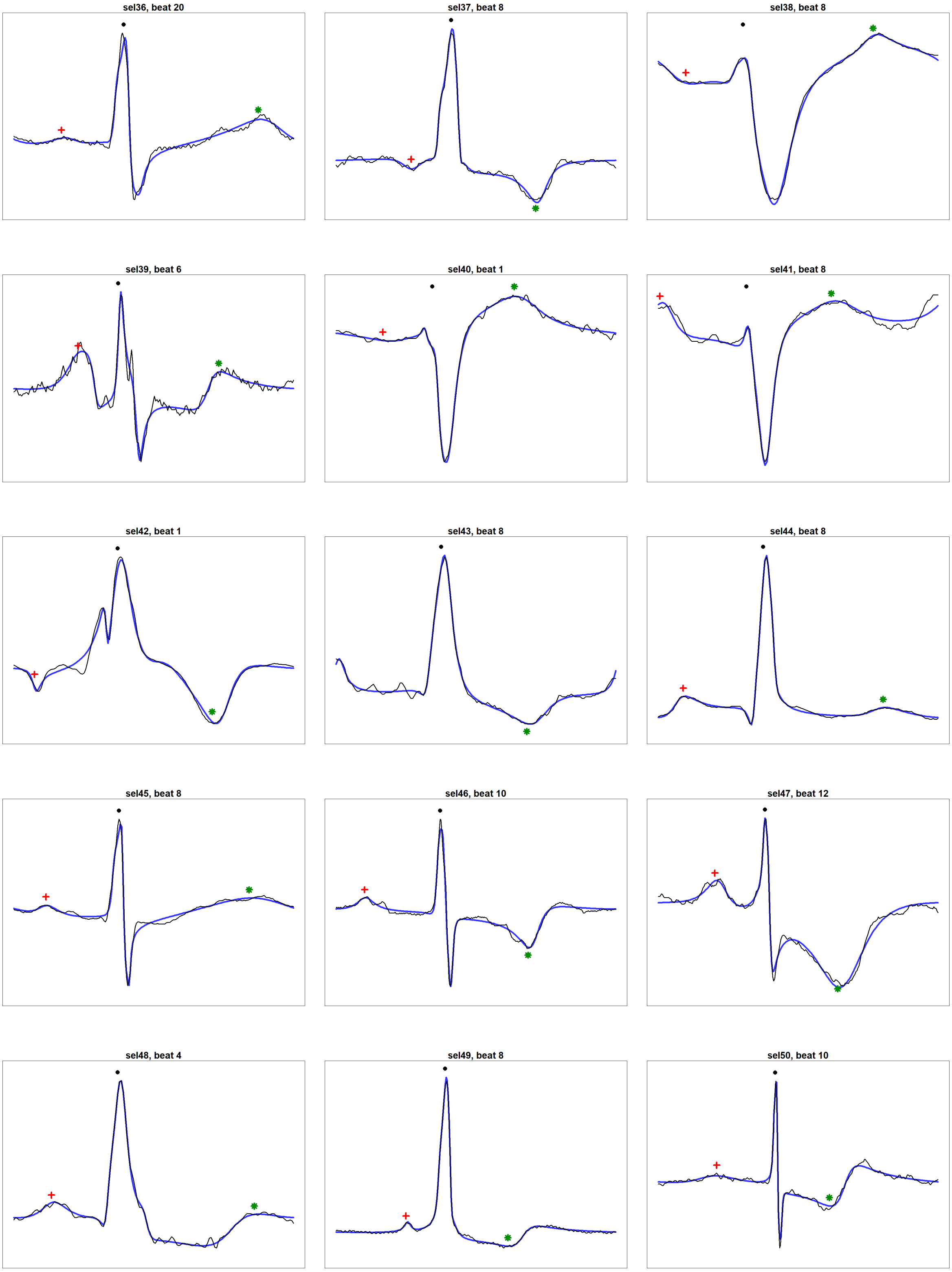

Figure S9: Representative beats (black line) for patients *sel36*, *sel37*, *sel38*, *sel39*, *sel40*, *sel41*, *sel42*, *sel43*, *sel44*, *sel45*, *sel46*, *sel47*, *sel48*, *sel49*, *sel50*; FMM_{ecg} fits (blue line) and fiducial marks for R wave(●), T wave($\star$), P wave($+$). Patients are sorted alphabetically

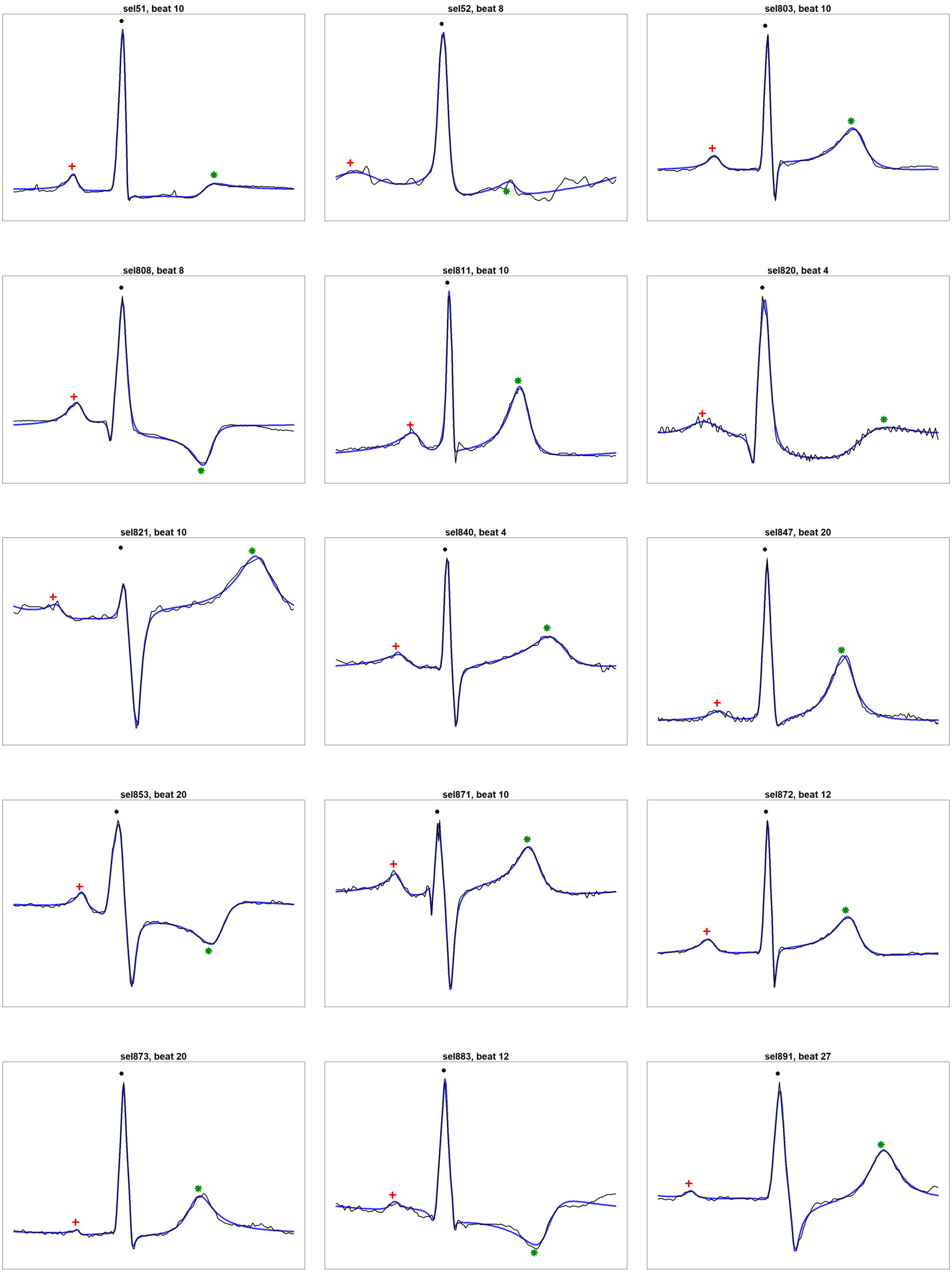

Figure S10: Representative beats (black line) for patients *sel51*,*sel52*, *sel803*, *sel808*, *sel811*, *sel820*, *sel821*, *sel840*, *sel847*, *sel853*, *sel871*, *sel872*, *sel873*, *sel883*, *sel891*; FMM_{ecg} fits (blue line) and fiducial marks for R wave(●), T wave($\star$), P wave(+). Patients are sorted alphabetically

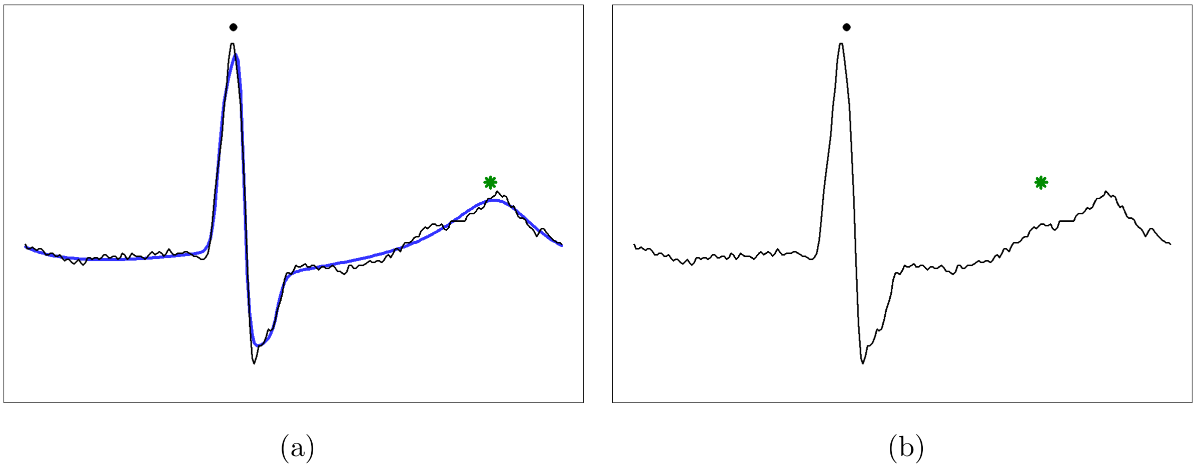

Figure S11: Patient *sel36*, beat 9. (a) FMM_{ecg} fit and annotations; (b) Physionet annotations; R wave (●), T wave ($\star$), P wave (+)

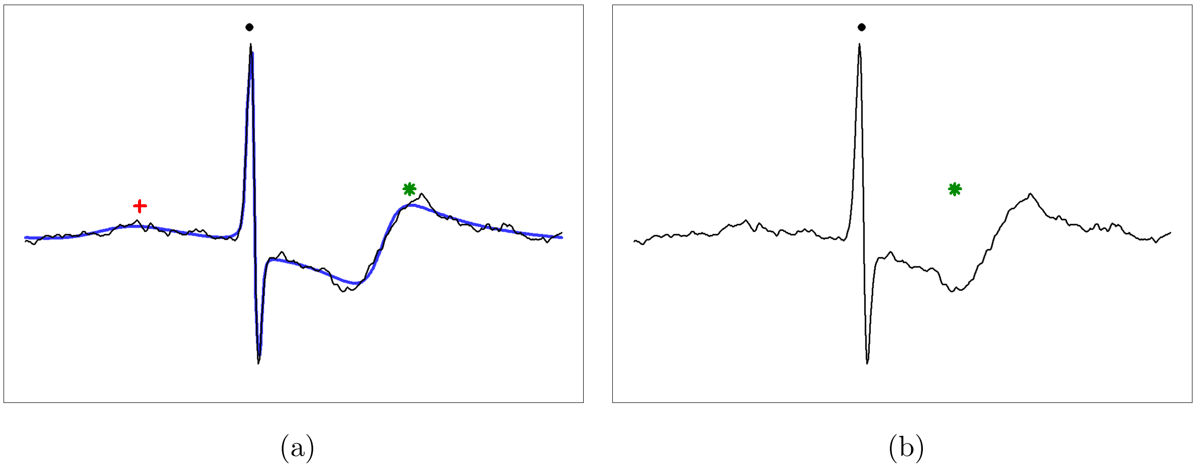

Figure S12: Patient *sel50*, beat 10. (a) FMM_{ecg} fit and annotations; (b) Physionet annotations; R wave (●), T wave ($\star$), P wave (+)

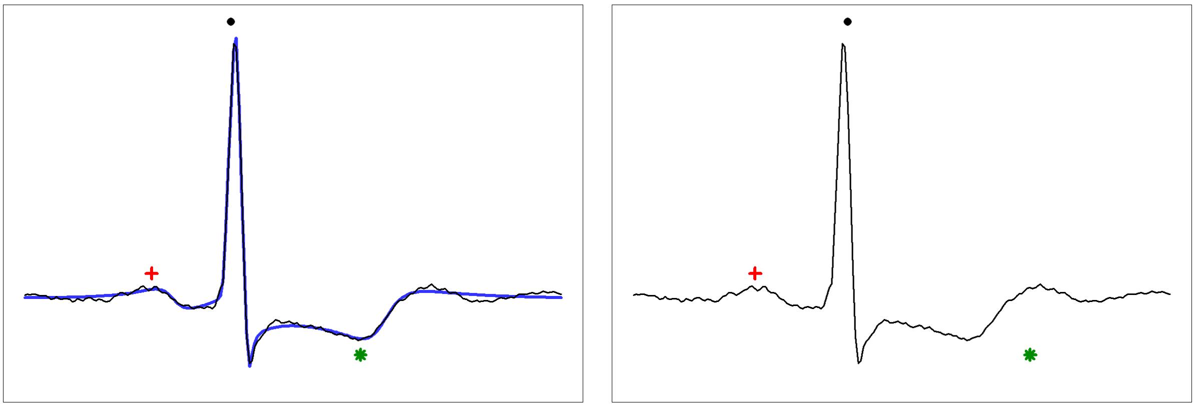

(a) (b)

Figure S13: Patient *sel301*, beat 8. (a) FMM_{ecg} fit and annotations; (b) Physionet annotations; R wave (•), T wave ($\star$), P wave (+)

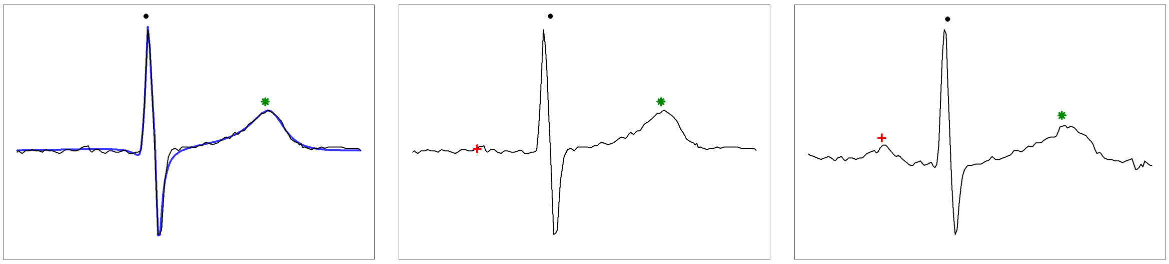

(a) (b) (c)

Figure S14: Patient *sel840*. (a) FMM_{ecg} fit and annotations, beat 1; (b) Physionet annotations, beat 1; (c) Physionet annotations, beat 4; R wave (•), T wave ($\star$), P wave (+)

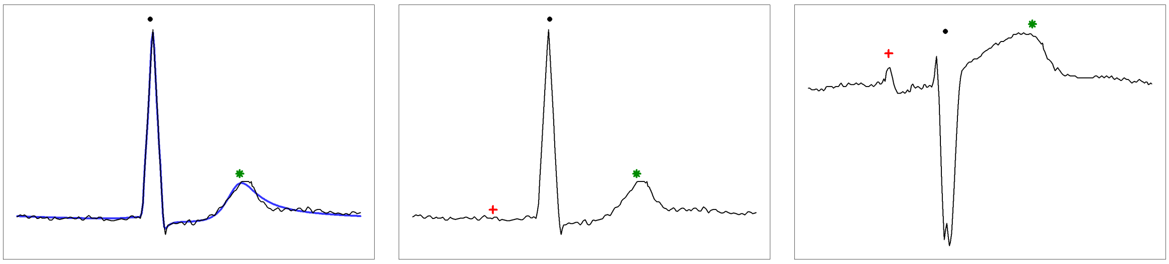

(a) (b) (c)

Figure S15: Patient *sel873*, beat 1. (a) FMM_{ecg} fit and annotations, signal 1; (b) Physionet annotations, signal 1; (c) Physionet annotations, signal 2; R wave (•), T wave ($\star$), P wave (+)

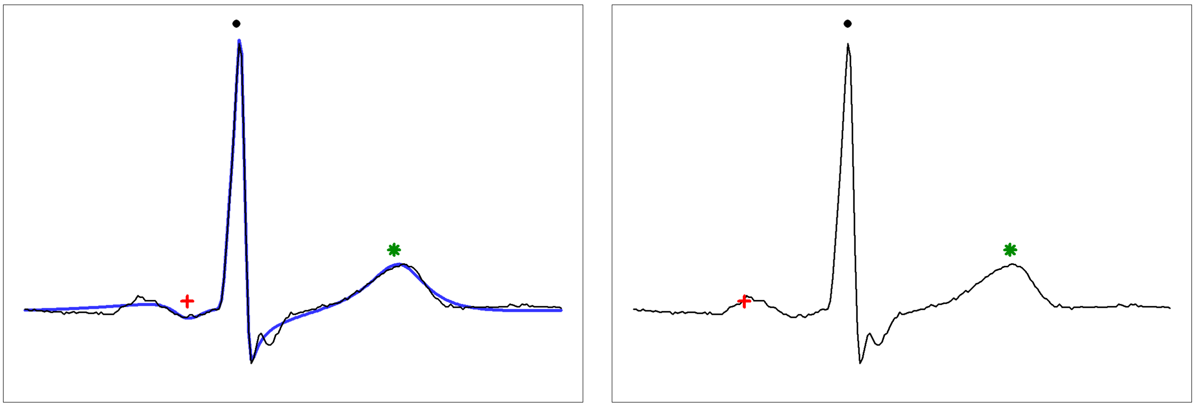

(a) (b)

Figure S16: Patient *sel09509*, beat 1. (a) FMM_{ecg} fit and annotations; (b) Physionet annotations; R wave (•), T wave ($\star$), P wave (+)

database. Finally, confusion matrices for QT subgroups patient identification analyses are shown from Tables S10 to S17.

Table S7: Diagonal values from Fisher LDA in QT database. Patients are sorted according to the subgroups given in Table S7

sel30	*sel31*	*sel32*	*sel33*	*sel34*	*sel35*	*sel36*	*sel37*	*sel38*	*sel39*	*sel40*	*sel41*	*sel42*	*sel43*	*sel44*
30	25	23	30	23	23	26	23	30	28	27	26	30	25	23
sel45	*sel46*	*sel47*	*sel48*	*sel49*	*sel50*	*sel51*	*sel52*	*sel100*	*sel102*	*sel103*	*sel104*	*sel114*	*sel116*	*sel117*
28	21	27	30	30	24	27	26	29	72	30	47	50	46	26
sel123	*sel213*	*sel221*	*sel223*	*sel230*	*sel231*	*sel232*	*sel233*	*sel301*	*sel302*	*sel306*	*sel307*	*sel308*	*sel310*	*sel803*
30	60	30	30	27	43	26	29	30	30	36	28	50	30	25
sel808	*sel811*	*sel820*	*sel821*	*sel840*	*sel847*	*sel853*	*sel871*	*sel872*	*sel873*	*sel883*	*sel891*	*sel0104*	*sel0106*	*sel0107*
30	30	30	24	56	33	29	56	23	29	26	46	30	30	34
sel0110	*sel0111*	*sel0112*	*sel0114*	*sel0116*	*sel0121*	*sel0122*	*sel0124*	*sel0126*	*sel0129*	*sel0133*	*sel0136*	*sel0166*	*sel0170*	*sel0203*
27	28	49	24	26	30	30	48	29	27	30	29	34	30	30
sel0210	*sel0211*	*sel0303*	*sel0405*	*sel0406*	*sel0409*	*sel0411*	*sel0509*	*sel0603*	*sel0604*	*sel0606*	*sel0607*	*sel0609*	*sel0612*	*sel0704*
30	30	30	29	28	30	30	30	27	27	15	30	30	29	24
sel14046	*sel14157*	*sel14172*	*sel15814*	*sel16265*	*sel16272*	*sel16273*	*sel16420*	*sel16483*	*sel16539*	*sel16773*	*sel16786*	*16795*	*sel17152*	*sel17453*
30	30	46	30	30	28	29	29	29	29	30	29	30	29	29

Table S8: QT subgroups description

Original Database	Size	Patient Label
Sudden Cardiac Death Holter	23	*sel30*, *sel31*, *sel32*, *sel33*, *sel34* , *sel35*, *sel36*, *sel37*, *sel38*, *sel39*, *sel40*, *sel41*, *sel42*, *sel43*, *sel44*, *sel45*, *sel46*, *sel47*, *sel48*, *sel49*, *sel50*, *sel51*, *sel52*
MIT BIH Arrhythmia	15	*sel100*, *sel102*, *sel103*, *sel104*, *sel114*, *sel116*, *sel117*, *sel123*, *sel213*, *sel221*, *sel223*, *sel230*, *sel231*, *sel232*, *sel233*
MIT BIH ST Change	6	*sel301*, *sel302*, *sel306*, *sel307*, *sel308*, *sel310*
MIT BIH Supraventricular Arrhythmia	13	*sel803*, *sel808*, *sel811*, *sel820*, *sel821*, *sel840*, *sel847*, *sel853*, *sel871*,*sel872*, *sel873*, *sel883*, *sel891*
European ST T	33	*sel0104*, *sel0106*, *sel0107*, *sel0110*, *sel0111*, *sel0112*, *sel0114*, *sel0116*, *sel0121*, *sel0122*, *sel0124*, *sel0126*, *sel0129*, *sel0133*, *sel0136*, *sel0166*, *sel0170*, *sel0203*, *sel0210*, *sel0211*, *sel0303*, *sel0405*, *sel0406*, *sel0409*, *sel0411*, *sel0509*, *sel0603*, *sel0604*, *sel0606*, *sel0607*, *sel0609*, *sel0612*, *sel0704*
MIT BIH Long Term	4	*sel14046*, *sel14157*, *sel14172*, *sel15814*
MIT BIH Normal Sinus Rhythm	11	*sel16265*, *sel16272*, *sel16273*, *sel16420* , *sel16483*, *sel16539*, *sel16773*, *sel16786*, *16795*, *sel17152* , *sel17453*

Table S9: Patient identification error rates in QT subgroups

Original Database	% Error Rate
Sudden Cardiac Death Holter	7.603
MIT BIH Arrhythmia	4.123
MIT BIH ST Change	0.556
MIT BIH Supraventricular Arrhythmia	3.249
European ST T	2.000
MIT BIH Long Term	1.306
MIT BIH Normal Sinus Rhythm	0.606

Table S10: Confusion matrix for Sudden Cardiac Death Holter QT subgroup

	sel30	*sel31*	*sel32*	*sel33*	*sel34*	*sel35*	*sel36*	*sel37*	*sel38*	*sel39*	*sel40*	*sel41*	*sel42*	*sel43*	*sel44*	*sel45*	*sel46*	*sel47*	*sel48*	*sel49*	*sel50*	*sel51*	*sel52*
sel30	30	0	0	0	0	0	0	0	0	0	0	0	0	0	0	0	0	0	0	0	0	0	0
sel31	2	28	0	0	0	0	0	0	0	0	0	0	0	0	0	0	0	0	0	0	0	0	0
sel32	3	0	27	0	0	0	0	0	0	0	0	0	0	0	0	0	0	0	0	0	0	0	0
sel33	0	0	0	30	0	0	0	0	0	0	0	0	0	0	0	0	0	0	0	0	0	0	0
sel34	0	0	0	0	29	1	0	0	0	0	0	0	0	0	0	0	0	0	0	0	0	0	0
sel35	0	0	1	0	1	26	0	0	0	0	0	0	0	0	2	0	0	0	1	0	0	0	0
sel36	0	0	0	0	0	0	27	1	0	0	0	0	0	0	0	0	1	0	1	0	0	0	1
sel37	4	0	2	1	0	0	0	35	0	0	0	0	0	0	0	0	1	0	0	0	0	5	2
sel38	0	0	0	0	0	0	0	0	30	0	0	0	0	0	0	0	0	0	0	0	0	0	0
sel39	0	0	0	0	0	0	0	0	0	28	0	0	0	0	0	0	1	0	1	0	0	0	0
sel40	0	0	0	0	0	0	0	0	3	0	27	0	0	0	0	0	0	0	0	0	0	0	0
sel41	0	0	0	0	0	0	0	0	0	0	1	29	0	0	0	0	0	0	0	0	0	0	0
sel42	0	0	0	0	0	0	0	0	0	0	0	0	30	0	0	0	0	0	0	0	0	0	0
sel43	0	0	4	0	0	0	0	0	0	0	0	0	0	26	0	0	0	0	0	0	0	0	0
sel44	0	0	0	0	0	2	0	0	0	0	0	0	0	0	27	0	0	0	1	0	0	0	0
sel45	0	0	0	0	0	0	0	0	0	0	0	0	0	0	0	29	0	0	0	1	0	0	0
sel46	0	0	0	0	0	0	0	1	0	3	0	0	0	0	0	0	25	0	0	0	0	1	0
sel47	0	0	0	0	0	0	0	0	0	0	0	0	0	0	0	0	0	30	0	0	0	0	0
sel48	0	0	0	0	0	0	0	0	0	0	0	0	0	0	0	0	0	0	30	0	0	0	0
sel49	0	0	0	0	0	0	0	0	0	0	0	0	0	0	0	0	0	0	0	30	0	0	0
sel50	0	0	0	0	1	0	0	0	0	0	1	0	0	0	0	1	1	0	0	0	28	0	0
sel51	0	0	0	0	0	0	2	0	0	0	0	0	0	0	0	0	0	0	0	0	0	28	0
sel52	0	0	1	0	0	0	0	1	0	0	0	0	0	0	1	0	0	0	0	1	0	0	26

Table S11: Confusion matrix for MIT BIH Arrhythmia QT subgroup

	sel100	*sel102*	*sel103*	*sel104*	*sel114*	*sel116*	*sel117*	*sel123*	*sel213*	*sel221*	*sel223*	*selsel230*	*sel231*	*sel232*	*sel233*
sel100	30	0	0	0	0	0	0	0	0	0	0	0	0	0	0
sel102	0	79	0	5	0	0	0	0	0	0	0	0	0	1	0
sel103	0	0	30	0	0	0	0	0	0	0	0	0	0	0	0
sel104	4	4	0	60	1	0	0	0	0	4	2	2	0	0	0
sel114	0	0	0	0	49	0	0	0	0	0	1	0	0	0	0
sel116	0	0	1	0	0	49	0	0	0	0	0	0	0	0	0
sel117	0	0	0	0	0	0	30	0	0	0	0	0	0	0	0
sel123	0	0	0	0	0	0	0	30	0	0	0	0	0	0	0
sel213	0	0	0	0	0	2	0	0	69	0	0	0	0	0	0
sel221	0	0	0	0	0	0	0	0	0	30	0	0	0	0	0
sel223	0	0	0	0	0	0	0	0	0	0	30	0	0	0	1
sel230	0	0	0	1	1	0	0	0	0	0	4	44	0	0	0
sel231	2	0	0	0	0	0	0	0	0	0	0	0	48	0	0
sel232	0	0	0	0	0	0	1	0	0	0	0	0	0	29	0
sel233	0	0	0	0	0	0	0	0	0	0	0	1	0	0	29

Table S12: Confusion matrix for MIT BIH ST Change QT subgroup

	sel301	*sel302*	*sel306*	*sel307*	*sel308*	*sel310*
sel301	30	0	0	0	0	0
sel302	0	30	0	0	0	0
sel306	0	0	36	0	0	0
sel307	0	1	0	29	0	0
sel308	0	0	0	0	50	0
sel310	0	0	0	0	0	30

Table S13: Confusion matrix for MIT BIH Supraventricular Arrhythmia QT subgroup

	sel803	*sel808*	*sel811*	*sel820*	*sel821*	*sel840*	*sel847*	*sel853*	*sel871*	*sel872*	*sel873*	*sel883*	*sel891*
sel803	26	0	0	0	0	0	0	0	0	4	0	0	0
sel808	0	30	0	0	0	0	0	0	0	0	0	0	0
sel811	0	0	30	0	0	0	0	0	0	0	0	0	0
sel820	0	0	0	30	0	0	0	0	0	0	0	0	0
sel821	0	0	0	0	30	0	0	0	0	0	0	0	0
sel840	2	0	0	0	0	68	0	0	0	0	0	0	0
sel847	0	0	0	0	0	0	33	0	0	0	0	0	0
sel853	0	0	0	0	0	0	0	30	0	0	0	0	0
sel871	0	0	0	0	0	0	0	0	68	0	0	0	2
sel872	4	0	0	0	0	0	0	0	0	26	0	0	0
sel873	0	0	0	0	0	0	0	0	0	0	33	0	0
sel883	0	0	0	0	0	0	0	0	0	0	0	30	0
sel891	0	0	0	0	0	0	0	0	6	0	0	1	64

Table S14: Confusion matrix for European ST T QT subgroup. Part I

	sel0104	*sel0106*	*sel0107*	*sel0110*	*sel0111*	*sel0112*	*sel0114*	*sel0116*	*sel0121*	*sel0122*	*sel0124*	*sel0126*	*sel0129*	*sel0133*	*sel0136*	*sel0166*	*sel0170*
sel0104	30	0	0	0	0	0	0	0	0	0	0	0	0	0	0	0	0
sel0106	0	30	0	0	0	0	0	0	0	0	0	0	0	0	0	0	0
sel0107	0	0	34	0	0	0	0	0	0	0	0	0	0	0	0	0	0
sel0110	0	0	0	27	0	0	0	0	0	0	0	0	0	0	0	0	0
sel0111	0	0	0	0	30	0	0	0	0	0	0	0	0	0	0	0	0
sel0112	0	0	0	0	0	50	0	0	0	0	0	0	0	0	0	0	0
sel0114	0	0	0	0	0	0	29	0	0	0	0	0	0	0	0	0	0
sel0116	0	0	0	0	0	0	0	28	0	0	0	0	0	0	0	0	0
sel0121	0	0	0	0	0	0	0	0	30	0	0	0	0	0	0	0	0
sel0122	0	0	0	0	0	0	0	0	0	30	0	0	0	0	0	0	0
sel0124	0	0	0	0	0	0	0	0	0	0	50	0	0	0	0	0	0
sel0126	0	0	0	0	0	0	0	0	0	0	1	29	0	0	0	0	0
sel0129	0	0	0	0	0	0	0	0	0	0	0	0	27	3	0	0	0
sel0133	0	0	0	0	0	0	0	0	0	0	0	0	0	30	0	0	0
sel0136	1	0	0	0	0	0	0	0	0	0	0	0	0	0	29	0	0
sel0166	0	0	0	0	0	0	0	0	1	0	0	0	0	0	0	35	0
sel0170	0	0	0	0	0	0	0	0	0	0	0	0	0	0	0	0	30

Table S15: Confusion matrix for European ST T QT subgroup. Part II

	sel0203	*sel0210*	*sel0211*	*sel0303*	*sel0405*	*sel0406*	*sel0409*	*sel0411*	*sel0509*	*sel0603*	*sel0604*	*sel0606*	*sel0607*	*sel0609*	*sel0612*	*sel0704*
sel0203	30	0	0	0	0	0	0	0	0	0	0	0	0	0	0	0
sel0210	0	30	0	0	0	0	0	0	0	0	0	0	0	0	0	0
sel0211	0	0	30	0	0	0	0	0	0	0	0	0	0	0	0	0
sel0303	0	0	0	30	0	0	0	0	0	0	0	0	0	0	0	0
sel0405	0	0	0	0	30	0	0	0	0	0	0	0	0	0	0	0
sel0406	0	0	0	0	0	30	0	0	0	0	0	0	0	0	0	0
sel0409	0	0	0	0	0	0	30	0	0	0	0	0	0	0	0	0
sel0411	0	0	0	0	0	1	0	29	0	0	0	0	0	0	0	0
sel0509	0	0	0	0	0	0	0	0	30	0	0	0	0	0	0	0
sel0603	0	0	0	0	0	0	0	1	0	27	0	0	0	0	0	0
sel0604	0	0	0	0	0	0	0	0	0	0	30	0	0	0	0	0
sel0606	0	0	0	0	0	0	0	0	0	0	0	30	0	0	0	0
sel0607	0	0	0	0	0	0	0	0	0	0	0	0	30	0	0	0
sel0609	0	0	0	0	0	0	0	0	0	0	0	0	0	30	0	0
sel0612	0	0	0	0	0	0	0	0	0	0	0	0	0	0	30	0
sel0704	0	0	0	0	1	0	0	1	0	0	0	0	0	0	0	27

Table S16: Confusion matrix for MIT BIH Long Term QT subgroup

	sel14046	*sel14157*	*sel14172*	*sel15814*
sel14046	30	1	0	0
sel14157	0	30	0	0
sel14172	1	0	49	0
sel15814	0	0	0	30

Table S17: Confusion matrix for MIT BIH Normal Sinus Rhythm QT subgroup

	sel16265	*sel16272*	*sel16273*	*sel16420*	*sel16483*	*sel16539*	*sel16773*	*sel16786*	*sel16795*	*sel17152*	*sel17453*
sel16265	30	0	0	0	0	0	0	0	0	0	0
sel16272	0	30	0	0	0	0	0	0	0	0	0
sel16273	1	0	29	0	0	0	0	0	0	0	0
sel16420	0	0	0	30	0	0	0	0	0	0	0
sel16483	0	0	0	0	29	0	0	0	1	0	0
sel16539	0	0	0	0	0	30	0	0	0	0	0
sel16773	0	0	0	0	0	0	30	0	0	0	0
sel16786	0	0	0	0	0	0	0	30	0	0	0
sel16795	0	0	0	0	0	0	0	0	30	0	0
sel17152	0	0	0	0	0	0	0	0	0	30	0
sel17453	0	0	0	0	0	0	0	0	0	0	30

References

[1] Bayes de Luna, A. *Basic Electrocardiography* (John Wiley & Sons, 2007).

[2] de Bliek, E. C. St elevation: Differential diagnosis and caveats. a comprehensive review to help distinguish st elevation myocardial infarction from nonischemic etiologies of st elevation. *Turkish journal of emergency medicine* **18**, 1–10 (2018).

[3] Hanna, E. B. & Glancy, D. L. St-segment depression and t-wave inversion: Classification, differential ddiagnosis, and caveats. *Cleveland Clinic journal of medicine* **78**, 404–414 (2011).

[4] Levis, J. T. Ecg diagnosis: Hyperacute t waves. *The Permanente journal* **19**, 79 (2015).

[5] Maršánová, L. *et al.* Ecg features and methods for automatic classification of ventricular premature and ischemic heartbeats: A comprehensive experimental study. *Scientific reports* **7**, 11239 (2017).

[6] Srivastva, R. & Singh, Y. N. Ecg analysis for human recognition using non-fiducial methods. *IET Biometrics* **8**, 295–305 (2019).
